# Complementarity and sensitivity of benthic state indicators to bottom‐trawl fishing disturbance

**DOI:** 10.1002/eap.3050

**Published:** 2024-10-12

**Authors:** P. Daniël van Denderen, Maider Plaza‐Morlote, Sandrine Vaz, Sander Wijnhoven, Angel Borja, Ulla Fernandez‐Arcaya, José M. González‐Irusta, Jørgen L. S. Hansen, Nikolaos Katsiaras, Andrea Pierucci, Alberto Serrano, Sofia Reizopoulou, Nadia Papadopoulou, Mattias Sköld, Christopher J. Smith, Henrik Nygård, Gert Van Hoey, Grete E. Dinesen, Elina A. Virtanen, Aurélien Boyé, Ana García‐Alegre, Juan Bellas, Stefan Bolam, Pablo Durán Muñoz, Mar Sacau, Giada Riva, Ellen Kenchington, Saša Raicevich, David Reid, Marie Julie Roux, Jan Geert Hiddink, Sebastian Valanko

**Affiliations:** ^1^ DTU Aqua Technical University of Denmark Kongens Lyngby Denmark; ^2^ Graduate School of Oceanography University of Rhode Island Narragansett Rhode Island USA; ^3^ IEO‐COST (CSIC), Centro Oceanográfico de Santander Instituto Español de Oceanografía, Centro Superior de Investigaciones Científicas Santander Spain; ^4^ MARBEC – Marine Biodiversity Exploitation and Conservation Univ Montpellier, CNRS, Ifremer, IRD Sète France; ^5^ Ecoauthor, Scientific Writing & Ecological Expertise Heinkenszand The Netherlands; ^6^ AZTI, Marine Research, Basque Research and Technology Alliance (BRTA) Pasaia Spain; ^7^ Department of Ecoscience Aarhus University Aarhus Denmark; ^8^ Hellenic Centre for Marine Research Institute of Oceanography Anavyssos Greece; ^9^ European Commission, Joint Research Centre Directorate D‐Sustainable Resources Ispra Italy; ^10^ Hellenic Centre for Marine Research Institute of Marine Biological Resources and Inland Waters Heraklion Crete Greece; ^11^ Institute of Marine Research Swedish University of Agricultural Sciences Lysekil Sweden; ^12^ Finnish Environment Institute (Syke) Helsinki Finland; ^13^ Flanders Research Institute of Agriculture, Fishery and Food Ostend Belgium; ^14^ Finnish Natural History Museum University of Helsinki Helsinki Finland; ^15^ Ifremer, DYNECO Plouzané France; ^16^ Centro Oceanográfico de Vigo Instituto Español de Oceanografía (IEO), CSIC Vigo Spain; ^17^ The Centre for Environment, Fisheries and Aquaculture Science Suffolk UK; ^18^ University of Padova Venice Italy; ^19^ Ocean and Ecosystem Science Division, Department of Fisheries and Oceans Bedford Institute of Oceanography Dartmouth Nova Scotia Canada; ^20^ ISPRA – Istituto Superiore per la Protezione e la Ricerca Ambientale, Area BIO‐CIT Chioggia Italy; ^21^ Marine Institute Oranmore Galway Ireland; ^22^ Maurice‐Lamontagne Research Institute Fisheries and Oceans Canada Mont‐Joli Quebec Canada; ^23^ School of Ocean Sciences Bangor University Menai Bridge UK; ^24^ International Council for the Exploration of the Sea Copenhagen Denmark

**Keywords:** benthos, biological traits, good environmental state, infauna, seabed integrity, seafloor habitats, soft sediments

## Abstract

Many indicators have been developed to assess the state of benthic communities and identify seabed habitats most at risk from bottom trawling disturbance. However, the large variety of indicators and their development and application under specific geographic areas and management contexts has made it difficult to evaluate their wider utility. We compared the complementarity/uniqueness, sensitivity, and selectivity of 18 benthic indicators to pressure of bottom trawling. Seventeen common datasets with broad regional representation covering a range of pressure gradients from bottom trawling disturbance (*n* = 14), eutrophication (*n* = 1), marine pollution (*n* = 1), and oxygen depletion (*n* = 1) were used for the comparison. The outcomes of most indicators were correlated to a certain extent with response to bottom trawling disturbance, and two complementary groups of indicators were identified: diversity‐based and biological trait‐based indicators. Trait‐based indicators that quantify the changes in relative abundance of sensitive taxa were most effective in identifying benthic community change in response to bottom trawling disturbance. None of the indicators responded to the trawling pressure gradient in all datasets, and some showed a response that were opposed to the theoretical expectation for some gradients. Indicators that showed clear responses to bottom trawling disturbance also showed clear responses in at least one other pressure gradient, suggesting those indicators are not pressure specific. These results emphasize the importance of selecting several indicators, at least one from each group (diversity and trait‐based), to capture the broader signals of change in benthic communities due to bottom trawling activities. Our systematic approach offers the basis from which scientific advisors and/or managers can select suitable combinations of indicators to arrive at a sensitive and comprehensive benthic status assessment.

## INTRODUCTION

Indicators that express the state of seabed ecosystems have been developed to assess changes in benthic diversity and community structure and function in response to human pressures (Borja et al., 2015; Diaz et al., 2004). Indicators provide a quantitative tool for scientists and policymakers, occasionally initiating further investigations or the basis upon which potential management measures may be formulated (Borja et al., 2012; Rice & Rochet, 2005). They are needed for the identification of limits, or thresholds, of how much change in benthic community structure can occur before seabed habitat integrity and ecological functions are compromised (Van Hoey et al., 2010).

Benthic indicators vary in their complexity, from easy‐to‐understand metrics, such as species richness, to measures of ecosystem function, to complex multi‐metric indicators (Birk et al., 2012). The number of benthic indicators that have been proposed is ever increasing (Borja et al., 2015; Diaz et al., 2004). There has been a growing interest in developing indicators for assessing the benthic community state in shelf seas (Rice et al., 2012) that are impacted by bottom trawls (Eigaard et al., 2017). Benthic fauna displays a variety of responses to bottom trawling disturbances, with most species and community‐level metrics showing declines, resulting in changes in the species and trait composition (Sciberras et al., 2018). In a meta‐analysis of 41 bottom‐trawl impact studies on shelf habitats, Hiddink et al. (2020) compared seven benthic community indicators and found that total abundance (number of individuals) and biomass showed the largest amount of change to bottom trawling. Other, single‐area, studies showed that indicator approaches tailored to biological traits can be more sensitive to trawling than whole community abundance and biomass (Jac et al., 2020; McLaverty et al., 2020; Serrano et al., 2022). Thus, a wide range of indicators are available that can be used to assess the state of seafloor habitats in relation to bottom trawling disturbances. However, the large variety of benthic state indicators, and differences in their geographic and management context specificities, has made it difficult to ultimately decide which indicator should be best adopted for evaluating progress toward management objectives for seabed habitats.

Assessing the usefulness of indicators can be facilitated by evaluating their complementarity, sensitivity, and specificity (ICES, 2022). Complementarity pertains to the distinctiveness of indicators and allows selecting a suite of indicators that each reflect variation in different attributes of the ecosystem component. The sensitivity of an indicator reflects the amount of change in response to changes in pressure intensity (Bundy et al., 2019). The change should be unambiguous and in a predictable direction, based on theoretical or empirical knowledge, and reflect the effect of a change in pressure on the state of the benthic community. The specificity of an indicator reflects whether it is primarily responsive to single or multiple pressures, whether this is considered desirable is generally context‐specific (Houle et al., 2012). “Specific” indicators are better at evaluating the efficacy of management actions by responding specifically to changes in the pressure or benthic community characteristic they are designed for. “Non‐specific” indicators meanwhile are considered desirable where the goal is to detect a change in benthic community status, for example, as an early warning, without necessarily attributing the change to a specific pressure.

Here, we evaluated the performance of 18 benthic indicators that are used to detect changes in benthic communities and assess adverse effects on seabed habitats. Using a common benthic dataset, we evaluated the performance of these indicators against three criteria: (1) The complementarity/uniqueness of each indicator in its response to pressure of bottom trawling; (2) the sensitivity of each indicator to pressure of bottom trawling; and (3) the specificity of each indicator to pressure of bottom trawling relative to three other anthropogenic pressure gradients, that is, a pollution, eutrophication, and oxygen‐depletion gradient.

## METHODS

### Pressure gradient datasets

**FIGURE 1:**
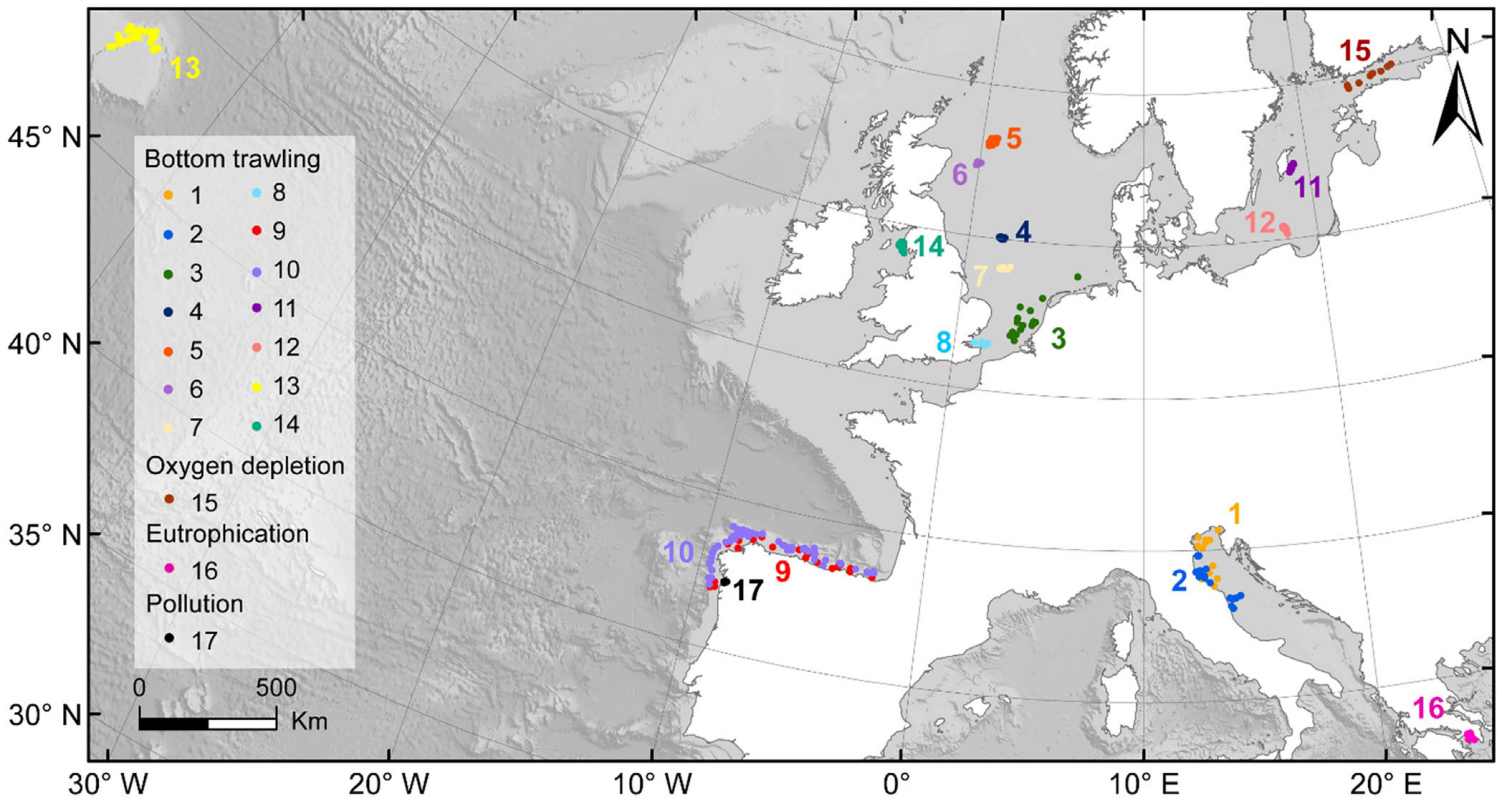
Map of the 17 benthic sampling gradients in the Baltic Sea, Atlantic Ocean, and Mediterranean Sea. Gradient numbers are identified in Table 1, colors are used to distinguish between gradients.

We compiled 17 benthic datasets: 14 that sampled benthic ecosystems over gradients of low to high commercial bottom trawling intensity, and one each that sampled over a gradient of eutrophication, oxygen depletion, and pollution (Table 1, Figure 1, Appendix S1). We focused on gradient datasets as these allow quantifying the effects of increasing human‐induced pressure intensity under realistic conditions. Ten of the gradients targeted a specific area (<50 × 50 km) and were designed to examine differences in benthic community composition along the gradient of human‐induced pressure intensity, while minimizing other environmental gradients such as depth, sediment type and bed shear stress. The other seven datasets were derived from benthic monitoring programs that covered larger spatial scales, without controlling for confounding environmental variables. For such datasets, we selected stations with matching sediment type and depth (see Appendix S1 for details) from the larger monitoring program and used these to evaluate indicators response. Samples were collected by trawls, grabs, or box cores (Table 1). In several datasets, replicate samples were taken at each sampling location (Appendix S1) and those data were summed.

**TABLE 1:** Description of the 17 datasets used to test indicator performance.

No.	Location	Sampling method	No. stations (replicate)	Sediment type	Depth range (m)	Max. distance (km)	Indicators not evaluated
Bottom trawl gradients							
1	Adriatic Sea—Italian EEZ	Rapido trawl	12 (1)	Sand	9–56	207	DKI
2	Adriatic Sea—Italian EEZ	Rapido trawl	16 (1)	Mud	8–87	233	DKI
3	North Sea—Dutch EEZ	Box core	15 (1)	Sand	22–36	329	SoS
4	North Sea—Dogger Bank	Hamon grab	7 (5)	Sand	25–30	20	
5	North Sea—Fladen Ground	Day grab	14 (5)	Mud	143–153	41	
6	North Sea—Long Forties	Hamon grab	5 (5)	Gravelly sand	74–83	19	
7	North Sea—Silver Pit	Box core	6 (4)	Muddy sand	68–78	40	
8	North Sea—Thames	Box core	6 (4)	Sand	16–40	49	
9	Northern Iberian Coast	Otter trawl	20 (1)	Sand	71–202	594	DKI
10	Northern Iberian Coast	Otter trawl	52 (1)	Mud	186–936	605	DKI
11	Baltic Sea—Gotland	van Veen grab	8 (1)	Muddy sand	37–59	35	TDI's, mT
12	Baltic Sea—Polish EEZ	Box core	11 (5)	Sand	70–85	32	
13	NW Atlantic—Flemish Cap	Otter trawl	26 (1)	Bathyal	786–1236	185	*D*_M_′, DKI, TDI's, mT, BENTIX, AMBI, M‐AMBI, A, *H*′, IS, SI
14	Irish Sea—Sellafield	Day grab	15 (5)	Muddy sand	21–42	42	SoS
Oxygen depletion gradient							
15	Gulf of Finland	van Veen grab	8 (3)	Mud	56–84	179	TDI's, mT
Eutrophication gradient							
16	Saronikos Gulf	Box core	8 (2)	Mixed sand/mud	20–94	33	
Pollution gradient of chemical contaminants							
17	Vigo Estuary	Box core	20 (1)	Mud	<30	13	Lf, Lm, TDI's, B, AMBI, M‐AMBI

*Note*: Further details are found in Appendix S1. Distance: Maximum distance between stations.

Abbreviation: EEZ, Exclusive Economic Zone.

### Benthic indicators

We calculated 18 benthic indicators (Table 2, Appendix S2). The general ecological indicators examined were whole community biomass B, abundance of individuals A, species richness R, Shannon–Wiener diversity *H*′, Simpson's dominance index SI, inverse Simpson IS and relative Margalef diversity *D*_M_′, which was modified from Margalef diversity by accounting for case‐specific reference diversity (Wijnhoven et al., 2022, see Appendix S2 for further information). Additionally, four indicators estimated an index score based on the sensitivity and diversity of a benthic community. AZTI's Marine Biotic Index (AMBI; Borja et al., 2000) estimates the proportion of individual abundance or biomass in five ecological groupings that are related to the degree of species sensitivity/tolerance to, primarily, anthropogenic‐induced changes in water quality and organic enrichment of sediments. A related multi‐metric indicator, Multivariate AMBI (M‐AMBI; Muxika et al., 2007), uses the AMBI score together with information on community diversity and richness. The BENTIX (Simboura & Zenetos, 2002) biotic index follows a comparable scoring as AMBI and estimates the ratio between two ecological groups of species (tolerant vs. sensitive taxa). The Danish Quality Index (DKI; Carstensen et al., 2014) uses the AMBI score for the community together with Shannon diversity, and the two indices are weighted equally and normalized to ambient salinity. Four indicators use biological trait information to score sensitivity: The Trawling Disturbance Index (TDI; de Juan & Demestre, 2012) uses traits to classify animals into five groups related to the degree of sensitivity/tolerance to trawling. The modified TDI (mTDI) is analogous to TDI and directly uses the TDI species score weighted by the relative biomass or abundance of the species to obtain an index (Jac et al., 2020). The Modified Vulnerability Index (mT) arises from a generic framework adapted to any kind of pressure and related traits, and uses the trait based TDI scores as well as the species protection status (Jac et al., 2020). Median Longevity (Lm) estimates the median longevity of a community based on the observation that, on average, long‐lived organisms are more vulnerable to trawling (Hiddink et al., 2018). Lastly, three indicators focused on the proportion of sensitive taxa in a community. The Partial TDI (pTDI) selects the most sensitive taxa based on the TDI score and estimates the abundance or biomass fraction relative to total biomass weighted by the TDI sensitivity score (Jac et al., 2020). The Sentinels of Seabed (SoS; Serrano et al., 2022) index estimates the biomass fraction of sensitive taxa relative to total biomass using different sensitivity classifications. For bottom trawling, SoS uses biological traits based on the BEnthos Sensitivity Index to Trawling Operations (BESITO) method (González‐Irusta et al., 2018) and grouped taxa. Because trait information was not available for all case studies, taxa were grouped according to their longevity as a replacement of BESITO in some case studies (Appendix S2). SoS selects the most sensitive taxa in the non‐trawl disturbance gradients using the AMBI groups. Long‐lived fraction (Lf) estimates the biomass fraction of the most long‐lived species in each community.

**TABLE 2:** Benthic state indicators used in the analyses with a brief description of each approach.

Benthic indicators (abbreviation)	Description
Abundance indicators	
Biomass (B)	Whole sampled community biomass
Abundance (A)	Whole sampled community no. individuals
Diversity indicators	
Richness (R)	No. unique species recorded in the sampled community
Relative Margalef diversity (*D*_M_′)	Species richness index that compensates for sample size and that is rescaled relative to reference conditions for good state (no or low‐pressure situation).
Shannon Index (*H*′)	Species richness index that measures how similar the abundances of different species are in a community
Simpson Index (SI)	Species evenness index that measures the effective no. species in a community
Inverse Simpson (IS)	Species evenness index based on the inverse of SI
Indicators with a diversity and sensitivity component	
AZTI's Marine Biotic Index (AMBI)	Index that estimates the proportions of individual abundance in five ecological groups, which are related to species sensitivity/tolerance to a pressure gradient
Multivariate AMBI (M‐AMBI)	Multi‐metric index composed of a sensitivity component based on AMBI and a diversity component represented by *H*′ and R
BENTIX Biotic Index (BENTIX)	The relative contribution of generally tolerant and sensitive taxa
Danish Quality Index (DKI)	Multi‐metric index composed of a sensitivity component based on AMBI and a diversity component represented by *H*′, both normalized to the salinity
Indicators with a trait‐based sensitivity component	
Trawling Disturbance Index (TDI)	Logarithmically weighted abundance index that classifies animals in five trait‐based groups, related to species sensitivity/tolerance to bottom trawling
Modified TDI (mTDI)	Index that classifies species based on a trait‐based sensitivity score to bottom trawling weighted by the abundance proportion of each species in the community
Modified vulnerability index (mT)	Index that distinguishing between direct and indirect effects of trawling and aggravating factors (factors that may increase sensitivity) using trait‐based scores
Median longevity (Lm)	Median life span of a benthic community based on cumulative community biomass
Sensitive taxa indicators	
Partial TDI (pTDI)	Index as mTDI, but only focused on the most sensitive trait‐based taxa
Sentinels of Seabed (SoS)	Biomass fraction of the most sensitive species in a community scored using trait‐based ecological groupings based on BESITO or AMBI depending on the pressure
Long‐lived fraction (Lf)	Biomass fraction of the most long‐lived species in a community

*Note*: Further details are found in Appendix S2.

Several indicators need reference conditions to calculate indicator values and the methodology is provided in Appendix S2. Not all indicators could be calculated for all gradients due to missing community abundance or biomass information and missing trait scores for dominant taxa (see Table 1, Appendix S2). In addition, indicator values for TDI, pTDI, mT, and mTDI were missing for some stations in some gradients due to partially available trait information. Moreover, several indicators can both be calculated with community abundance or biomass and Appendix S2 compares the abundance versus biomass‐based values of some of these indicators. Lastly, AMBI and mT were reversed in all analyses (by multiplying with minus one and adding the maximum initial value) as low values indicate a good state and high values a poor state. The reversed AMBI and mT indicators are termed AMBI and mT throughout the manuscript.

### Complementarity of indicators to trawling

We computed the Spearman correlation coefficients between indicators within each bottom‐trawl gradient to examine complementarity in responses. Average correlation across all trawl gradients was plotted by ordering indicators with Ward's hierarchical clustering. The number of observations to compute the correlations vary across indicator pairs depending on data availability (see Appendix S3 for average values and SDs).

### Sensitivity of indicators to trawling

The sensitivity of indicators to trawling was tested using two methods: (1) A meta‐analysis approach that estimated the mean response of each indicator trawling disturbance by comparing stations with low and high trawling intensity, and (2) a linear regression approach that estimated the change in indicator values along each trawl gradient using all sampling stations. Since trawling intensity was expressed in a variety of ways, direct comparisons at the same pressure value were not conducted and we avoided discussing specific trawling intensity values.

For the meta‐analysis approach, we computed the mean of each indicator at stations with low and high trawling intensity within each gradient. The selection of low‐fished stations involved choosing all those with a swept area ratio (SAR) below 0.35 per year (Appendix S4: Table S1). This value was chosen as previous work indicates that European shelf habitats experiencing these trawling intensities are likely to reflect, or are close to, undisturbed reference conditions (Bolam et al., 2017). Sampling stations in gradient number 14 were all above this SAR value and we therefore selected the two least‐fished stations. Within the three gradients lacking SAR values, we selected all zero‐fished stations within gradient numbers 8 and 13 and the two least‐fished stations in gradient number 7. Conversely, the selection of high trawling intensity stations involved selecting, where data permitted, the five most heavily fished stations. In instances where fewer than five high‐trawling intensity stations were available, we included all stations with SAR values more than 1 per year (see Appendix S4: Table S1). For gradient numbers 7 and 8 with few sampling stations and without SAR values, the three most heavily fished stations were selected. Subsequently, we verified the reliability of the results to station selection (Appendix S4: Figure S1).

The analysis of the mean response was conducted using a weighted meta‐analysis approach via linear mixed‐effects models in the r package “metafor” (Viechtbauer, 2010). The response variable for the candidate indicator *I* was expressed as the log response‐ratio, denoted as ln(*I*_trawled_/*I*_control_), representing the logarithm of the ratio between the mean indicator values at the most heavily fished stations *I*_trawled_ and the least fished stations *I*_control_ (following Hiddink et al., 2020 and references therein). Gradient datasets were weighted by the inverse of variance of the original study taking SD and number of observations in the low and the high groups into account. Two indicator methods (SoS and Lf) had near zero *I*_trawled_ values, which were adjusted to 0.01 to prevent inflated log response‐ratios and CIs. The TDI‐based indicators had missing values in some gradients but were included since they had information from at least two stations for both low and high trawling treatment. The mean response to trawling was also separately estimated for samples collected by (1) bottom trawls that mainly sample large epifauna and megafauna, and (2) grabs/cores that mainly sample small epifauna and infauna.

The linear regression approach was used to estimate the change in each indicator along each trawl gradient. The predictor variable, trawling intensity, was log10 (× + 1)‐transformed as we expected most indicators to decline exponentially with trawling (Sciberras et al., 2018). We verified that similar results were obtained with a more flexible generalized additive model (GAM) (not shown). A statistical model with trawling intensity was seen as most parsimonious when the Akaike information criterion (AIC) was lower than the null model, which was a model without trawling intensity. The TDI‐based indicators were retained in the linear regression analysis of each gradient when they had information at both low and high intensity and for at least half of the stations in each gradient.

### Specificity of indicators

We assessed the specificity of each indicator to pressure of bottom trawling intensity by evaluating indicator response to three non‐trawl pressure types, that is, pollution, eutrophication, and oxygen‐depletion. This evaluation was not intended to be a comprehensive analysis of each of these pressures but rather to explore whether certain indicators have a higher specificity to pressure of bottom trawling than others.

We estimated the effect of eutrophication, oxygen depletion, and pollution (Table 1) on each indicator with a GAM. The eutrophication gradient in the Saronikos Gulf has estimated the % of organic carbon and total nitrogen in the sediment at each sampling location. Since there is a correlation of 0.98 between total nitrogen and organic carbon, we selected one variable (total nitrogen) to reflect pressure intensity. Oxygen depletion in the Gulf of Finland is based on measured dissolved oxygen concentration 1 m above the seafloor at time of sampling. Pollution in the Vigo estuary is based on a cumulative pollution index that combines several contaminants (e.g., cadmium, mercury) in one index (Bellas et al., 2011). As with trawling, we compared the model with the pressure against a null model.

## RESULTS

### Complementarity of indicators to trawling

**FIGURE 2:**
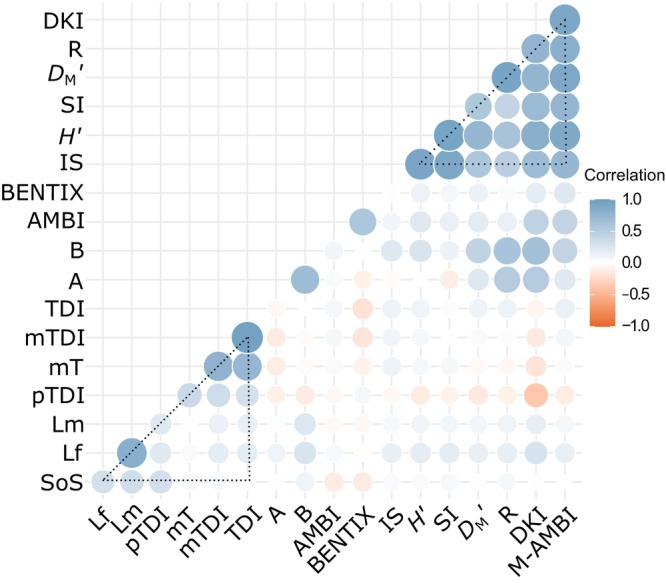
Complementarity of indicators based on the average correlation of indicators across all trawling gradients. Correlations were ordered with Ward's hierarchical clustering. Triangles were added to support visual interpretation. For abbreviations, see Table 2.

Most indicators are correlated to a certain extent, and two clustered groups of indicators can be recognized (Figure 2). In these clusters, indicators are more correlated with each other than with indicators outside those clusters. The first group encompasses diversity‐based indicators (richness R, *D*_M_′, *H*′, SI and IS) and multi‐metric indicators with a diversity and sensitivity component (M‐AMBI and DKI). A second group comprises indicators relying on biological traits. Within this group, the TDI‐based indicators (TDI, mTDI, mT, and pTDI) are highly correlated. SoS, Lm, Lf, and pTDI also display strong correlations, which is understandable, as three of these indicators estimate the proportion of taxa sensitive to trawling. AMBI and BENTIX strongly correlate with each other, moderately with the diversity indicators and weakly with most of the trait‐based indicators. Community abundance and biomass are highly correlated with each other, show a moderate positive correlation with the diversity indicators, and are negatively correlated with most TDI‐based indicators. BENTIX, community abundance, and pTDI most often have a negative correlation with the other indicators. Several correlated indicators show a high variability in the correlations between gradient datasets (Appendix S3: Table S2).

### Sensitivity of indicators to trawling

**FIGURE 3:**
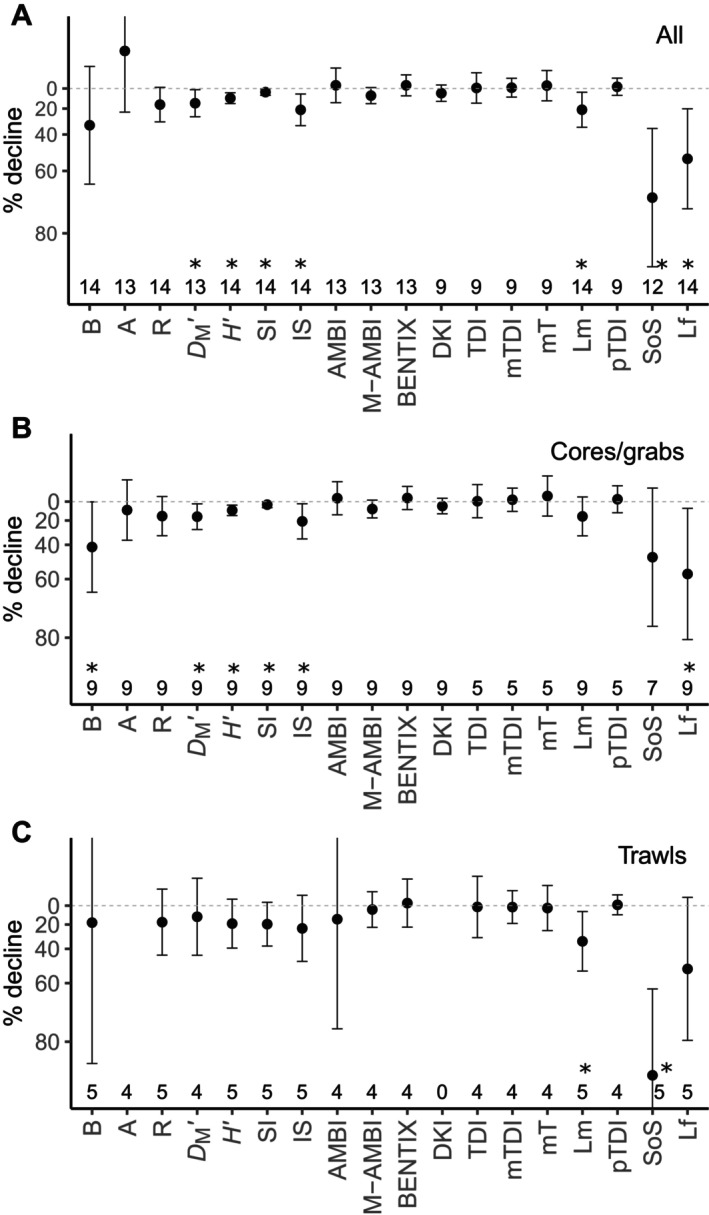
Response (mean with 95% CIs) of benthic indicators to bottom trawling disturbance (A) and separately for data collected with cores and grabs (B) and trawls (C). A significant effect of trawling is marked with an *. The numbers above the *x*‐axis show the number of gradient datasets used. Abundance in (C) is much higher than zero and not shown. For abbreviations, see Table 2.

Most indicators either show a decline at stations with highest trawling pressure relative to baseline stations with lowest intensity, or no response (Figure 3A). The largest declines are seen in SoS and Lf, two indicators that target the biomass of sensitive fauna in the community. Both SoS and Lf show an average decline that is larger than 50% in the given trawling gradients. Biomass shows an average decline of 40%, but has large CIs, whereas richness, *D*_M_′, *H*′, IS and Lm all show smaller declines (15%–20%) but more consistent responses. The abundance of individuals A is higher in trawl‐disturbed areas. A significant difference between indicator values at low and high trawling is present when the 95% CIs do not overlap with zero, and this is the case for *D*_M_′, *H*′, SI, IS, Lm, SoS, and Lf.

**FIGURE 4:**
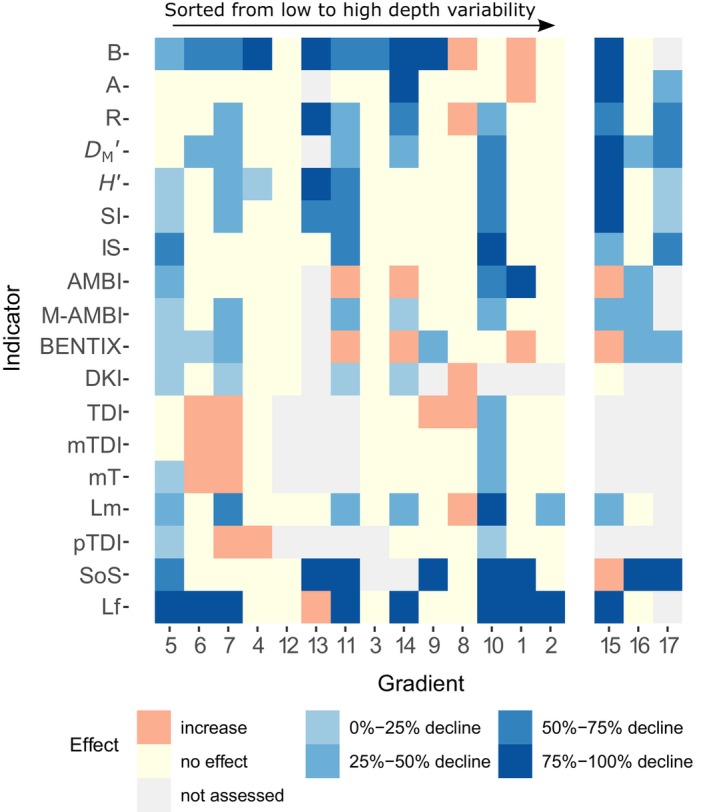
Relative response of each indicator as a function of each pressure gradient (gradient numbers match Figure 1 and Table 1). The trawl disturbance gradients are sorted from low to high depth variability based on the CV of depth. The effect is estimated as the predicted change in indicator value from the lowest to highest pressure intensity in each gradient. The effect is defined as “no effect” when the Akaike information criterion (AIC) of the model without the pressure is equal or lower than the AIC of the model with the pressure. For abbreviations, see Table 2.

Qualitatively similar patterns are observed for most indicators between grab/core samples and trawl samples (Figure 3B,C). Abundance shows the largest difference as it is showing no significant difference between low and high trawling sites in the grab/core subset, while abundance is 10× higher in high trawl‐intensity sites relative to baseline stations in the trawl gear subset (not visible in Figure 3B). This large difference is mainly driven by one gradient dataset (Italian EEZ sand, gradient number 1 in Table 1) where abundance of individuals is 33× higher in the high intensity sites (Figure 4). Aside from abundance, only Lm and SoS show a significant difference between low and high trawling stations in the trawl gear subset, while biomass, *D*_M_′, *H*′, IS, SI, and Lf show a significant difference between low and high trawling stations in the grab/core subset (Figure 3B,C).

**FIGURE 5:**
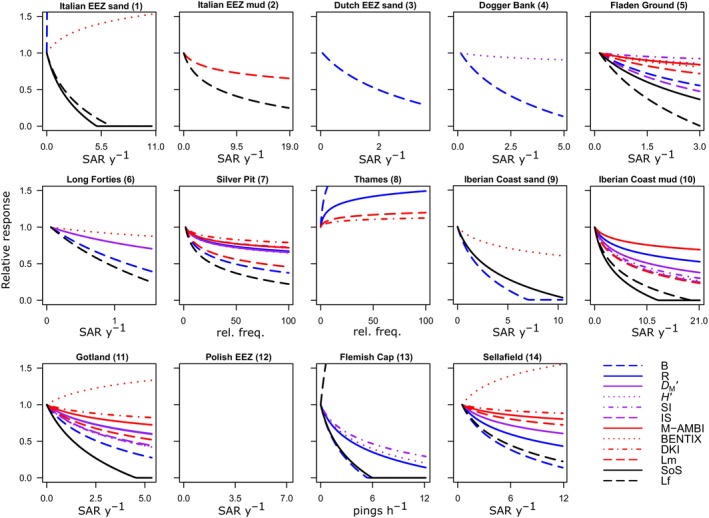
Relative response of each indicator as a function of trawling intensity. Lines are fitted with a linear model where trawling intensity is log10(*x* + 1) transformed. Lines are included when the model with trawling has a lower Akaike information criterion than the null model. Names and numbers above each plot correspond to Table 1. Only indicators that were significantly declining in at least four gradient datasets are included (all outputs are shown in Figure 4). No indicator changes were found in the Polish Exclusive Economic Zone gradient. For abbreviations, see Table 2. Trawling intensity is measured as a relative frequency on a linear scale in gradient numbers 7 and 8, as number of Vessel Monitoring by Satellite pings per square kilometer in number 13 and as swept area ratio (SAR) per year in all other gradients.

The indicators that declined in most trawl disturbance gradients are biomass B (64% of the gradients), Lf (57%), and SoS (50%) (Figure 4). As observed in the meta‐analysis, the sensitive taxa indicators are declining most strongly in response to trawling in many gradient studies (Figures 4 and 5). None of the indicators shows a decline in gradients 8 (North Sea—Thames) and 12 (Baltic Sea—Polish EEZ), which are both locations with mainly opportunistic taxa (see further discussion). At least half of the indicators show a decline with trawling in gradients 5, 7, 10, 11, and 14, which all had sampled on mud or muddy‐sand sediments (Table 2). Several indicators (SoS, *H*′, M‐AMBI, *D*_M_′, SI, and IS) show either a negative response or no response but were never observed to increase along the trawling gradient (Figures 4 and 5). Most TDI indicators increased or showed no response. Community biomass, abundance, and BENTIX increased with increasing pressure at the four locations with largest depth variability (Figure 4).

### Specificity of indicators to trawling

**FIGURE 6:**
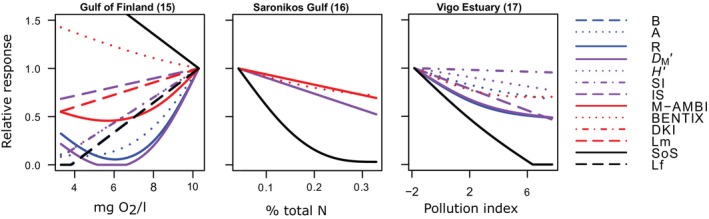
Relative response of each indicator as a function of oxygen, % of total nitrogen and pollution. Lines are fitted with a generalized additive model and included when the model with the pressure has a lower Akaike information criterion than the null model. Names and numbers above each plot correspond to Table 1. We included all available indicators, except for AMBI which is not shown to avoid plotting the reverse of AMBI (see *Methods*). For abbreviations, see Table 2.

The indicators that show a strong decline in relation to trawling show a less consistent response to pollution, oxygen depletion, and eutrophication. SoS, which is now based on sensitive taxa using the AMBI groups, shows the strongest decline in the pollution and eutrophication gradient, but increases in the oxygen depletion gradient. *D*_M_′ shows the most consistent decline across all three gradients (Figures 4 and 6). M‐AMBI shows a clear decline in the oxygen depletion and eutrophication gradient.

## DISCUSSION

Our study explored eighteen methodologies for calculating indicators, each potentially responsive to bottom trawling pressure. Our findings reveal that only some indicator methods consistently establish a pressure–response relationship, demonstrating their effectiveness. In contrast, various other indicators, though routinely applied in specific regions, lack the consistent evidence required for broader applicability. Yet, none of the indicators were responsive to trawling in all studied gradients. This highlights that assessments of benthic community change will be enhanced by capitalizing on the strengths of different methodologies and combining complementary indicators.

### Sensitivity of indicators to trawling

We found that methods that quantify the fraction of sensitive taxa in the community (such as in SoS or Lf) offer the best capability to effectively identify benthic community change with increasing bottom trawling pressure. While it is unsurprising that sensitive taxa demonstrate increased responsiveness, the challenge is defining what sensitive taxa are. The three indicators that focused on sensitive taxa were all built upon methods that used a biological trait‐based approach to select sensitive taxa. Of these, Lf, using trait information on longevity, and SoS, using a mixture of biological traits (González‐Irusta et al., 2018), were most successful across the diverse datasets. These methods are likely to be useful for monitoring benthic state in other geographic regions. Both longevity in Lf and some of the traits used in SoS (e.g., feeding mode, longevity) are not directly related to trawl selectivity, as are traits such as mobility and flexibility. However, longevity is a trait that affects recoverability (Hiddink et al., 2018; Rijnsdorp et al., 2018) and this is likely a reason why these indicators are responsive to chronic trawling disturbance.

Several of the remaining indicators, that is, richness, *D*_M_′, *H*′, IS, and Lm, displayed a consistent but modest decline to trawling across the various gradient studies. A previous meta‐analysis, including four of the datasets analyzed here, suggested that community abundance and biomass have the most desirable properties for assessing benthic communities disturbed by bottom trawls (Hiddink et al., 2020). Our analysis confirms community biomass sensitivity to trawling with declines in 75% of the trawl gradients, yet the response was less consistent across the gradient studies. Both community biomass and abundance were also found to increase in some trawl gradients. It is noticeable that these increases were only found in datasets that covered major depth gradients or large spatial scales, and confounding of the pressure with environmental drivers might explain such increases. However, such an increase can also occur when fish and invertebrate predators are reduced by fishing, and when this reduction in predation mortality outweighs the increase in trawling mortality in the remaining benthic community (van de Wolfshaar et al., 2020). Such an increase has been previously observed in some trawling impact studies (González‐Irusta et al., 2018; Serrano et al., 2022; Sköld et al., 2018) and Serrano et al. (2022) suggested that where total biomass is used as an indicator, it can best be accompanied by functional information on the sensitivity of the groups that contribute to that biomass.

### Complementarity of indicators to trawling

We identified two complementary clusters of indicators: those based on diversity and those reliant on traits. This distinction suggests that various components within benthic communities may exhibit differing responses to trawling pressure. It underscores the significance of selecting multiple indicators, at least one from each cluster, to ensure the inclusion of diversity components, species sensitivity and community abundance in assessments. Such a combined approach can both amplify the sensitivity and consistency of indicator‐based assessments as well as provide a more holistic representation of benthic community change, as has earlier been suggested for other anthropogenic pressures, for example, Van Hoey et al. (2010).

The use of a suite of indicators may also support the identification of areas that are most vulnerable to pressure of bottom trawling. In several gradients, many indicators declined with trawling, thereby indicating that several community aspects are impacted. These gradients all reflected mud or muddy‐sand sediments, supporting previous findings showing that muddy sediments can be more sensitive to trawling than sand (Sciberras et al., 2018). In European shelf waters, muddy sediments are the most heavily fished substrate type (Eigaard et al., 2017) and muddy benthic communities are likely to experience, or have already adapted to, high levels of trawl disturbance and benthic impact. Conversely, some gradients (numbers 3 and 8 in the southern North Sea and number 12 in the Polish EEZ) had only a few declining indicators. Previous work in these areas showed that all have opportunistic taxa at the least disturbed sampling sites, likely the result of a high tidal bed stress in gradient numbers 3 and 8 and relatively low bottom oxygen concentrations and salinity in gradient number 12 (van Denderen et al., 2015, 2022). Our results suggest that these opportunistic taxa are also more resistant to trawl disturbance. One can, however, debate whether “low pressure” stations in gradients with relatively high occurrence of opportunistic taxa reflect low pressure situations or whether other pressures play a role in benthic habitat quality in these areas. In the latter case, our study has mainly tested the current additional impact of trawling on the benthic communities.

### Specificity of indicators to trawling

Indicators that showed clear responses to bottom‐trawl disturbance also declined in at least one of the three other, non‐trawling, pressure gradients (i.e., eutrophication, pollution, and oxygen depletion), and no obvious differences in specificity to pressure of bottom trawling were detected. On the other hand, some indicators, effective in detecting eutrophication and pollution impacts, demonstrated limited responsiveness to bottom trawling (AMBI, DKI, and BENTIX), supporting the findings by McLaverty et al. (2023). These indicators may thus be more effective in detecting and monitoring the impacts of diffuse pressures. However, our evaluation of specificity was not meant to be exhaustive and further analyses are needed to examine more subtle differences in specificity.

### Use of gradient studies

Examining gradients of commercial bottom trawling intensity poses a challenge, as observed variations in benthic communities along the trawling gradient may mistakenly appear to be correlated with trawling intensity. Approximately half of the gradient studies were specifically designed to limit the risk of confounding factors by minimizing environmental gradients. The other gradients were sampled as part of a monitoring program across larger areas, potentially across a range of environments, thereby increasing the potential for confounding effects. To address this, we selected stations with matching sediment type and depth from the larger monitoring program. An alternative approach to manage these confounding factors could involve incorporating the environmental variables in the models and extracting only the marginal effect of trawling. However, this method could lead to underestimating the total effect of trawling if it covaries with certain environmental variables that have a limited impact on the benthic community. For that reason, we decided to use a subset of the data. This decision was also guided by previous research indicating that bottom trawling often has larger impacts on benthic communities than relatively small changes in environmental conditions (Jac et al., 2022; Tillin et al., 2006; van Denderen et al., 2015).

Since most trawl‐sampled gradients were part of a monitoring program, it remains unclear whether differences between the trawl and grab/core subset are driven by sampling design or type of fauna sampled. Further clarification could be achieved by conducting additional trawl‐sampled gradient studies as well as estimating the effect of environmental drivers on community composition in unimpacted areas (Bolam et al., 2017).

## CONCLUSIONS

We have delineated a systematic approach for utilizing diverse indicator methods in a manner that could facilitate scientific advisors and/or managers to make robust and transparent policy decisions regarding bottom trawling. Based on the selectivity and complementarity outcomes of our study, we propose the use of trait‐based indicators that quantify the changes in relative abundance of sensitive taxa, such as SoS, or Lf. The application of such indicators should be complemented by the simultaneous use of methods that compute indices that address biodiversity aspects, such as *H*′ and *D*_M_′, and whole community biomass. This combined assessment will ensure capturing the signals of change in benthic communities and seafloor integrity due to bottom trawling activities. Although the ultimate selection of benthic indicators is contingent upon policy objectives for biodiversity conservation and sustainable use, our study informs the choice of methods used to track change in relation to management measures. It allows decision‐making to examine the evidence supporting effective indicator methodologies in a transparent and informed manner.

## AUTHOR CONTRIBUTIONS

All authors reviewed and revised the manuscript critically. P. Daniël van Denderen led the analysis and the writing of the paper with input from Ellen Kenchington, Saša Raicevich, David Reid, Marie Julie Roux, Jan Geert Hiddink, and Sebastian Valanko. Maider Plaza‐Morlote, Sandrine Vaz, Sander Wijnhoven, Angel Borja, Ulla Fernandez‐Arcaya, José González‐Irusta, Jørgen Hansen, Nikolaos Katsiaras, Andrea Pierucci, Alberto Serrano, Sofia Reizopoulou, and P. Daniël van Denderen estimated indicators and wrote indicator methods. Ana García‐Alegre, Juan Bellas, Stefan Bolam, Pablo Durán Muñoz, Henrik Nygård, Mar Sacau, Giada Riva, José González‐Irusta, Nikolaos Katsiaras, Sofia Reizopoulou, Saša Raicevich, Jan Geert Hiddink, and P. Daniël van Denderen prepared gradient datasets and wrote gradient methods.

## CONFLICT OF INTEREST STATEMENT

The authors declare no conflicts of interest.

## Supporting information


Appendix S1:

Appendix S2:

Appendix S3:

Appendix S4:

## Data Availability

Data (van Denderen et al., 2023) are available in Zenodo at https://doi.org/10.5281/zenodo.8308127. Code (van Denderen, 2024) is available on Zenodo at https://doi.org/10.5281/zenodo.10880101
