# Supplementary material for: Complementarity and sensitivity of benthic state indicators to bottom‐trawl fishing disturbance
Source: Ecol Appl. 2024 Oct 12;34(8):e3050. doi: 10.1002/eap.3050 (PMC11610696; doi:10.1002/eap.3050)
Supplement: Supplementary file 1 — Appendix S1: [file EAP-34-e3050-s004.pdf]

Ecological Applications

Appendix S1 – Metadata for common dataset

Complementarity and sensitivity of benthic state indicators to bottom-trawl fishing disturbance

P. Daniël van Denderen, Maider Plaza-Morlote, Sandrine Vaz, Sander Wijnhoven, Angel Borja, Ulla Fernandez-Arcaya, José M. González-Irusta, Jørgen L. S. Hansen, Nikolaos Katsiaras, Andrea Pierucci, Alberto Serrano, Sofia Reizopoulou, Nadia Papadopoulou, Mattias Sköld, Christopher J Smith, Henrik Nygård, Gert Van Hoey, Grete E. Dinesen, Elina A. Virtanen, Aurélien Boyé, Ana García-Alegre, Juan Bellas, Stefan Bolam, Pablo Durán Muñoz, Mar Sacau, Giada Riva, Ellen Kenchington, Saša Raicevich, David Reid, Marie Julie Roux, Jan Geert Hiddink, Sebastian Valanko

- **Gradient number: 1**

Area: Adriatic Sea – Italian EEZ
Habitat Type: Circalittoral sand
Depth Range: 9-56 m
Sampling Gear: Rapido trawl
Sampling Program: Italian bottom trawl survey (SoleMon); all stations in the circalittoral sand habitat type were selected for the analysis.
Number of stations (samples per station): 12 (1)
Maximum distance between stations (km): 207
Sampling year (month): 2016 (11)
Contact for further information: Saša Raicevich (sasa.raicevich@isprambiente.it)
Reference: Riva (2022)

Dominant bottom fisheries in area, if any	Pressure intensity unit	Method to estimate pressure gradient	Biomass unit	Abundance unit	Environmental information
Beam and otter trawls	SAR per year average from 2012 to 2016	Sum of OTB and TBB (VMS and AIS data) (Russo et al. 2020)	Kg wet weight per km^2	Numbers per km^2	Depth and net primary production

- **Gradient number: 2**

Area: Adriatic Sea – Italian EEZ
Habitat Type: Circalittoral mud
Depth Range: 8-87 m
Sampling Gear: Rapido trawl
Sampling Program: Italian bottom trawl survey (SoleMon); all stations in the circalittoral mud habitat type were selected for the analysis.
Number of stations (samples per station): 16 (1)
Maximum distance between stations (km): 233
Sampling year (month): 2016 (11)
Contact for further information: Saša Raicevich (sasa.raicevich@isprambiente.it)
Reference: Riva (2022)

Dominant bottom fisheries in area, if any	Pressure intensity unit	Method to estimate pressure gradient	Biomass unit	Abundance unit	Environmental information
Beam and otter trawls	SAR per year average from 2012 to 2016	Sum of OTB and TBB (VMS and AIS data) (Russo et al. 2020)	Kg wet weight per km^2	Numbers per km^2	Depth and net primary production

- **Gradient number: 3**

Area: North Sea – Dutch EEZ “high tidal stress area”
Habitat Type: Sand
Depth Range: 22-36 m
Sampling Gear: Box core (0.078 m^2)
Sampling Program: Dutch infauna sampling program (MWTL); stations were selected based on similar depth and grainsize (see van Denderen et al. 2014)
Number of stations (samples per station): 15 (1)
Maximum distance between stations (km): 329
Sampling year (month): 2007 (3)
Contact for further information: Daniel van Denderen (pdvd@aqua.dtu.dk)
Reference: van Denderen et al. (2015)

Dominant bottom fisheries in area, if any	Pressure intensity unit	Method to estimate pressure gradient	Biomass unit	Abundance unit	Environmental information
Beam and otter trawls	SAR per year - last 365 days	Estimated from interpolated VMS data from Dutch fisheries on 0.001 x 0.001 grid	Gram ash-free dry weight per 0.078 m^2	Numbers per 0.078 m^2	Sediment type, depth, tidal bed stress

- **Gradient number: 4**

  Area: North Sea – Dogger Bank
  Habitat Type: Sand
  Depth Range: 25-30 m
  Sampling Gear: Hamon grab (0.1 m^2)
  Sampling Program: Scientific cruise
  Number of stations (samples per station): 7 (5)
  Maximum distance between stations (km): 20
  Sampling year (month): 2003 (9)
  Contact for further information: Jan Geert Hiddink (j.hiddink@bangor.ac.uk)
  Reference: Queirós et al. (2006)

Dominant bottom fisheries in area, if any	Pressure intensity unit	Method to estimate pressure gradient	Biomass unit	Abundance unit	Environmental information
Beam and otter trawls	SAR per year	Queirós et al. (2006)	Gram wet weight per 0.5 m^2	Numbers per 0.5 m^2	Sediment type, depth, tidal bed stress

- **Gradient number: 5**
  Area: North Sea – Fladen Ground
  Habitat Type: Mud
  Depth Range: 143−153 m
  Sampling Gear: Day grab (0.1 m^2)
  Sampling Program: Scientific survey
  Number of stations (samples per station): 14 (5)
  Maximum distance between stations (km): 41
  Sampling year (month): 2004 (6)
  Contact for further information: Jan Geert Hiddink (j.hiddink@bangor.ac.uk)
  Reference: Tillin et al. (2006)

**Dominant bottom fisheries in area, if any**	**Pressure intensity unit**	**Method to estimate pressure gradient**	**Biomass unit**	**Abundance unit**	**Environmental information**
Nephrops otter trawl	SAR per year	See Tillin et al. (2006)	Gram wet weight per 0.5 m^2	Numbers per 0.5 m^2	Sediment type, depth, tidal bed stress

- **Gradient number: 6**
  Area: North Sea – Long Forties
  Habitat Type: Gravelly sand
  Depth Range: 74−83 m
  Sampling Gear: Hamon grab (0.1 m^2)
  Sampling Program: Scientific cruise
  Number of stations (samples per station): 5 (5)
  Maximum distance between stations (km): 19
  Sampling year (month): 2003 (9)
  Contact for further information: Jan Geert Hiddink (j.hiddink@bangor.ac.uk)
  Reference: Tillin et al. (2006)

**Dominant bottom fisheries in area, if any**	**Pressure intensity unit**	**Method to estimate pressure gradient**	**Biomass unit**	**Abundance unit**	**Environmental information**
Scallop dredge	SAR per year	See Tillin et al. (2006)	Gram wet weight per 0.5 m^2	Numbers per 0.5 m^2	Sediment type, depth, tidal bed stress

- **Gradient number: 7**
  Area: North Sea – Silver Pit
  Habitat Type: Muddy sand
  Depth Range: 68–78 m
  Sampling Gear: Box corer (0.078 m^2)
  Sampling Program: Scientific cruise
  Number of stations (samples per station): 6 (4)
  Maximum distance between stations (km): 40
  Sampling year (month): 2002 (7)
  Contact for further information: Stefan Bolam (stefan.bolam@cefas.co.uk)
  Reference: van Denderen et al. (2015); selection of sampling stations and pressure intensity estimates are described in Jennings et al. (2001)

Dominant bottom fisheries in area, if any	Pressure intensity unit	Method to estimate pressure gradient	Biomass unit	Abundance unit	Environmental information
Beam and otter trawls	Relative frequencies of trawling disturbance on a linear scale	Estimated from aerial survey data collected by fisheries inspection services	gram wet weight per 0.31 m2	Numbers per 0.31 m^2	Sediment type, depth, tidal bed stress

- **Gradient number: 8**
  Area: North Sea – Thames
  Habitat Type: Sand
  Depth Range: 16–40 m
  Sampling Gear: Box corer (0.078 m^2)
  Sampling Program: Scientific cruise
  Number of stations (samples per station): 6 (4)
  Maximum distance between stations (km): 49
  Sampling year (month): 2002 (7)
  Contact for further information: Stefan Bolam (stefan.bolam@cefas.co.uk)
  Reference: van Denderen et al. (2015)

Dominant bottom fisheries in area, if any	Pressure intensity unit	Method to estimate pressure gradient	Biomass unit	Abundance unit	Environmental information
Beam and otter trawls	Relative frequencies of trawling disturbance on a linear scale	Estimated from aerial survey data collected by fisheries inspection services	gram wet weight per 0.31 m2	Numbers per 0.31 m^2	Sediment type, depth, tidal bed stress

- **Gradient number: 9**
  Area: Northern Iberian Coast
  Habitat Type: Sand (Offshore circalitoral sand)
  Depth Range: 71-202 m
  Sampling Gear: Otter trawl
  Sampling Program: Spanish IBTS (DEMERSALES); all stations in the offshore circalitoral sand habitat type were selected for the analysis.
  Number of stations (samples per station): 20 (1)
  Maximum distance between stations (km): 594
  Sampling year (month): 2016 (9 – 10)
  Contact for further information: José Manuel Gonzalez (jmanuel.gonzalez@ieo.csic.es)
  Reference: Serrano et al. (2022)

**Dominant bottom fisheries in area, if any**	**Pressure intensity unit**	**Method to estimate pressure gradient**	**Biomass unit**	**Abundance unit**	**Environmental information**
Otter trawl	SAR per year - average over the last 5 years	Estimated from interpolated VMS data from Spanish fisheries and calculated as the sum of the area swept by cell and year	Gram wet weight per km^2	Numbers per km^2	Depth

- **Gradient number: 10**
  Area: Southern Bay of Biscay/Northern Iberian Coast
  Habitat Type: Several, but mainly mud (upper bathyal sediment)
  Depth Range: 186-936 m
  Sampling Gear: Otter trawl
  Sampling Program: Spanish IBTS (DEMERSALES); all stations in the upper bathyal habitat type were selected for the analysis.
  Number of stations (samples per station): 52 (1)
  Maximum distance between stations (km): 605
  Sampling year (month): 2016 (9 – 10)
  Contact for further information: José Manuel Gonzalez (jmanuel.gonzalez@ieo.csic.es)
  Reference: Serrano et al. (2022)

**Dominant bottom fisheries in area, if any**	**Pressure intensity unit**	**Method to estimate pressure gradient**	**Biomass unit**	**Abundance unit**	**Environmental information**
Otter trawl	SAR per year - average over the last 5 years	Estimated from interpolated VMS data from Spanish fisheries and calculated as the sum of the area swept by cell and year	Gram wet weight per km^2	Numbers per km^2	Depth

- **Gradient number: 11**
  Area: Baltic Sea – Gotland
  Broad Habitat Type: Muddy sand
  Depth Range: 37-59 m
  Sampling Gear: van Veen grab (0.1 m^2)
  Sampling Program: Swedish benthic sampling program
  Number of stations (samples per station): 8 (1)
  Maximum distance between stations (km): 35
  Sampling year (month): 2012 (5)
  Contact for further information: Mattias Sköld (mattias.skold@slu.se)
  Reference: van Denderen et al. (2020)

Dominant bottom fisheries in area, if any	Pressure intensity unit	Method to estimate pressure gradient	Biomass unit	Abundance unit	Environmental information
Otter trawl	SAR per year based on the last year pre-sampling	Estimated from interpolated VMS data from Swedish and Danish fisheries and calculated as the sum of the area swept by trawls within a 250 m radius at each sampling location	Gram wet weight per 0.1 m^2	Numbers per 0.1 m^2	Sediment type and depth

- **Gradient number: 12**
  Area: Baltic Sea – Polish EEZ
  Habitat Type: Sand
  Depth Range: 70-85 m
  Sampling Gear: Box core (0.06 m^2)
  Sampling Program: Scientific cruise
  Number of stations (samples per station): 11 (5)
  Maximum distance between stations (km): 32
  Sampling year (month): 2018 (9)
  Contact for further information: Daniel van Denderen (pdvd@aqua.dtu.dk)
  Reference: van Denderen et al. (2022)

Dominant bottom fisheries in area, if any	Pressure intensity unit	Method to estimate pressure gradient	Biomass unit	Abundance unit	Environmental information
Otter trawl	SAR per year - average of 2012-2018	ICES VMS at 0.05 x 0.05 resolution (data call year is 2019)	Gram wet weight per 0.3 m^2	Numbers per 0.3 m^2	Sediment type, depth and bottom oxygen concentration

- **Gradient number: 13**
  Area: NW Atlantic – Flemish Cap
  Habitat Type: Mainly mud (bathyal sediment)
  Depth Range: 786-1236 m
  Sampling Gear: Lofoten bottom trawl
  Sampling Program: Scientific survey
  Number of stations (samples per station): 26 (1)
  Maximum distance between stations (km): 185
  Sampling year (month): 2007 (6-7)
  Contact for further information: Mar Sacau (mar.sacau@ieo.csic.es); Pablo Durán Muñoz (pablo.duran@ieo.csic.es)
  Reference: Murillo et al. (2016, 2020), Muñoz et al. (2020)

**Dominant bottom fisheries in area, if any**	**Pressure intensity unit**	**Method to estimate pressure gradient**	**Biomass unit**	**Abundance unit**	**Environmental information**
Otter trawls	Number of pings by square km	Estimated from VMS data from international fisheries and calculated as the sum of pings by cell and year	Gram wet weight per km^2	n/a	Depth

- **Gradient number: 14**
  Area: Irish Sea – Sellafield
  Habitat Type: Muddy sand
  Depth Range: 21−42 m
  Sampling Gear: Day grab (0.1 m^2)
  Sampling Program: Scientific cruise
  Number of stations (samples per station): 15 (5)
  Maximum distance between stations (km): 42
  Sampling year (month): 2009 (6)
  Contact for further information: Jan Geert Hiddink (j.hiddink@bangor.ac.uk)
  Reference: Hinz et al. (2009); Hiddink et al. (2011).

**Dominant bottom fisheries in area, if any**	**Pressure intensity unit**	**Method to estimate pressure gradient**	**Biomass unit**	**Abundanc e unit**	**Environmental information**
Otter trawl	SAR per year	See Hiddink et al. (2011)	Gram weight per 0.5 m^2	Numbers per 0.5 m^2	Sediment type, depth, tidal bed stress

- **Gradient number: 15**
  Area: Gulf of Finland
  Habitat Type: Circalittoral mud/Offshore circalittoral mud
  Depth Range: 56–84 m
  Sampling Gear: van Veen grab (0.112 m^2)
  Sampling Program: Finnish monitoring program
  Number of stations (samples per station): 8 (3)
  Maximum distance between stations (km): 179
  Sampling year (month): 2015 (5)
  Contact for further information: Henrik Nygård (henrik.nygard@syke.fi)
  Reference: Zoobenthos information system POHJE, Finnish Environment Institute (Syke). https://ckan.ymparisto.fi/dataset/pohjaelaintietojarjestelma-pohje

Dominant bottom fisheries in area, if any	Pressure intensity unit	Method to estimate pressure gradient	Biomass unit	Abundance unit	Environmental information
n/a	mg O_2/l	Measured dissolved oxygen concentration (1 meter above seafloor)	Gram wet weight per m^2 (converted from 0.33m^2)	Numbers per m^2 (converted from 0.33m^2)	Depth, sediment type, nutrients and hydrography

- **Gradient number: 16**
  Area: Saronikos Gulf
  Habitat Type: Mixed sand / mud
  Depth Range: 20-94 m
  Sampling Gear: Box corer (0.1 m^2)
  Sampling Program: Saronikos sampling programme 2012
  Number of stations (samples per station): 8 (2)
  Maximum distance between stations (km): 33
  Sampling year (month): 2012 (2)
  Contact for further information: Sofia Reizopoulou (sreiz@hcmr.gr)
  Reference: Pavlidou et al. (2019)

Dominant bottom fisheries in area, if any	Pressure intensity unit	Method to estimate pressure gradient	Biomass unit	Abundance unit	Environmental information
n/a	% total N	Data/pressure indicators: % of orgC and total N in the sediment at each sampling location. Pressure is based on total N (correlates with orgC)	Mg dry weight per 0.2 m^2	Numbers per 0.2 m^2	Sediment type, %org C, total N and depth

- **Gradient number: 17**
  Area: Vigo Estuary
  Habitat Type: Infralitoral Mud
  Depth Range: <30 m
  Sampling Gear: BOUMA box-corer (0.0175 m^2)
  Sampling Program: Pollution monitoring program
  Number of stations (samples per station): 20 (1)
  Maximum distance between stations (km): 13
  Sampling year (month): 2004-2006
  Contact for further information: Juan Bellas (juan.bellas@ieo.csic.es)
  Reference: Bellas et al. (2011), Beiras et al. (2012)

**Dominant bottom fisheries in area, if any**	**Pressure intensity unit**	**Method to estimate pressure gradient**	**Biomass unit**	**Abundance unit**	**Environmental information**
n/a	Cumulative pollution index	The CPI index combine several pollutants (e.g. Cd, Hg) in one index. See Bellas et al. (2011) for a complete description of CPI method	n/a	Numbers per km^2 (converted to km^2 from the core data)	Details on specific pollutants are available

References

Beiras, R., Durán, I., Bellas, J., Sánchez-Marín, P. (2012) Biological effects of contaminants: Paracentrotus lividus sea urchin embryo test with marine sediment elutriates. ICES Techniques in Marine Environ-mental Sciences, No. 51, 13 pp. DOI: http://dx.doi.org/10.25607/OBP-262

Bellas, J., Nieto, Ó., Beiras, R. (2011). Integrative assessment of coastal pollution: development and evaluation of sediment quality criteria from chemical contamination and ecotoxicological data. Continental Shelf Research, 31(5), 448–456.

Hinz, H., Prieto, V., Kaiser, M.J. (2009). Trawl disturbance on benthic communities: chronic effects and experimental predictions. Ecological Applications, 19(3), 761–773.

Hiddink, J.G., Johnson, A.F., Kingham, R. and Hinz, H. (2011), Could our fisheries be more productive? Indirect negative effects of bottom trawl fisheries on fish condition. Journal of Applied Ecology, 48: 1441-1449.

Jennings, S., Dinmore, T. A., Duplisea, D.E., Warr, K. J., Lancaster, J.E. (2001). Trawling disturbance can modify benthic production processes. Journal of Animal ecology, 70(3), 459–475.

Muñoz, P.D., Sacau, M., García-Alegre, A., Román, E. (2020). Cold-water corals and deep-sea sponges by-catch mitigation: Dealing with groundfish survey data in the management of the northwest Atlantic Ocean high seas fisheries. Marine Policy, 116, 103712.

Murillo, F.J., Serrano, A., Kenchington, E., Mora, J. (2016). Epibenthic assemblages of the Tail of the Grand Bank and Flemish Cap (northwest Atlantic) in relation to environmental parameters and trawling in-tensity. Deep Sea Research Part I: Oceanographic Research Papers, 109, 99–122.

Murillo, F.J., Weigel, B., Bouchard Marmen, M., Kenchington, E. (2020). Marine epibenthic functional di-versity on Flemish Cap (north-west Atlantic)—Identifying trait responses to the environment and map-ping ecosystem functions. Diversity and Distributions, 26(4), 460–478.

Pavlidou, A., Simboura, N., Pagou, K., Assimakopoulou, G., Gerakaris, V., Hatzianestis, I., ... Borja, A. (2019). Using a holistic ecosystem-integrated approach to assess the environmental status of Saronikos Gulf, Eastern Mediterranean. Ecological Indicators, 96, 336–350.

Queirós, A.M., Hiddink, J.G., Kaiser, M.J., Hinz, H. (2006). Effects of chronic bottom trawling disturbance on benthic biomass, production and size spectra in different habitats. Journal of Experimental Marine Biology and Ecology, 335(1), 91–103.

Riva, G. 2022. Applicazione e confronto di indici per la valutazione degli impatti della pesca a strascico demersale sulle comunità epibentoniche dell'Adriatico. MSc Thesis in Marine Biology. Università di Padova. Supervisors: C. Mazzoldi, S. Raicevich. Pp. 106.

Russo E., Monti M. A., Mangano M. C., Raffaetà A., Sarà G., Silvestri C., Pranovi F. (2020). Temporal and spatial patterns of trawl fishing activities in the Adriatic Sea (Central Mediterranean Sea, GSA17). Ocean and Coastal Management 192, 105231.

Serrano, A., de la Torriente, A., Punzón, A., Blanco, M., Bellas, J., Durán-Muñoz, P., ... González-Irusta, J. M. (2022). Sentinels of Seabed (SoS) indicator: Assessing benthic habitats condition using typical and sensitive species. Ecological Indicators, 140, 108979.

Tillin, H.M., Hiddink, J.G., Jennings, S., Kaiser, M.J. (2006). Chronic bottom trawling alters the functional composition of benthic invertebrate communities on a sea-basin scale. Marine Ecology Progress Series, 318, 31–45.

van Denderen, P.D., Hintzen, N.T., Rijnsdorp, A.D., Ruardij, P., van Kooten, T (2014). Habitat-specific effects of fishing disturbance on benthic species richness in marine soft sediments. Ecosystems, 17, 1216–1226.

van Denderen, P.D., Bolam, S.G., Hiddink, J.G., Jennings, S., Kenny, A., Rijnsdorp, A.D., Van Kooten, T. (2015). Similar effects of bottom trawling and natural disturbance on composition and function of ben-thic communities across habitats. Marine Ecology Progress Series, 541, 31–43.

van Denderen, P.D., Bolam, S.G., Friedland, R., Hiddink, J.G., Noren, K., Rijnsdorp, A.D., ... Valanko, S. (2020). Evaluating impacts of bottom trawling and hypoxia on benthic communities at the local, habi-tat, and regional scale using a modelling approach. ICES Journal of Marine Science, 77(1), 278–289.

van Denderen, P.D., Törnroos, A., Sciberras, M., Hinz, H., Friedland, R., Lasota, R., ... Hiddink, J.G. (2022). Effects of bottom trawling and hypoxia on benthic invertebrate communities. Marine Ecology Progress Series, 694, 13–27.
