# Supplementary material for: Complementarity and sensitivity of benthic state indicators to bottom‐trawl fishing disturbance
Source: Ecol Appl. 2024 Oct 12;34(8):e3050. doi: 10.1002/eap.3050 (PMC11610696; doi:10.1002/eap.3050)
Supplement: Supplementary file 2 — Appendix S2: [file EAP-34-e3050-s003.pdf]

Ecological Applications

Appendix S2 – Calculating indicator values

Complementarity and sensitivity of benthic state indicators to bottom-trawl fishing disturbance

P. Daniël van Denderen, Maider Plaza-Morlote, Sandrine Vaz, Sander Wijnhoven, Angel Borja, Ulla Fernandez-Arcaya, José M. González-Irusta, Jørgen L. S. Hansen, Nikolaos Katsiaras, Andrea Pierucci, Alberto Serrano, Sofia Reizopoulou, Nadia Papadopoulou, Mattias Sköld, Christopher J Smith, Henrik Nygård, Gert Van Hoey, Grete E. Dinesen, Elina A. Virtanen, Aurélien Boyé, Ana García-Alegre, Juan Bellas, Stefan Bolam, Pablo Durán Muñoz, Mar Sacau, Giada Riva, Ellen Kenchington, Saša Raicevich, David Reid, Marie Julie Roux, Jan Geert Hiddink, Sebastian Valanko

A description of each indicator method is provided below. This description includes information on how indicator values were estimated from the common data set.

Section S1 M-AMBI and AMBI
Section S2 DKI - Danish Quality Index
Section S3 Relative Margalef diversity (D_M')
Section S4 BENTIX
Section S5 SoS – Sentinels of the Seabed
Section S6 mTDI, pTDI, TDI and mT
Section S7 Median longevity and fraction long-lived organisms
Section S8 Biomass, Abundance, Species Richness, Shannon Index, Inverse Simpson, Simpson Index
Section S9 Overview of indicators

Section S1: M-AMBI and AMBI

AMBI was developed to assess the status of benthic macroinvertebrates under different human pressures (e.g. pollution, eutrophication, aquaculture, dredging, etc.) and to manage the impacts they produce. Further, M-AMBI was developed to be compliant with the requirements of the European Water Framework Directive, and to assess the status of macroinvertebrates under different pressures.

AMBI was estimated from both abundance and biomass data. To calculate AMBI, cephalopods and one fish species were removed since the method is for macroinvertebrates. Also, some records at a very high taxonomic level (e.g., ‘bivalves’) were removed, following the guidelines for its use (Borja and Muxika, 2005). AMBI includes an expert-based species library with >12,000 macrobenthic species from where the sensitivity of local/regional species is drawn.

M-AMBI needs reference conditions, which are associated with the area and characteristics of the habitat (depth, grain size, community, etc.). To obtain reference conditions, some of the gradients were merged to larger similar geographical areas, but only in cases where both the sampling size and the units were the same (e.g. DB-FG-SEL-PH, SP-TH, which are assumed to have the same reference conditions). M-AMBI was afterwards calculated for each geographical region using abundance data. Calculations of M-AMBI were made using as the ‘best reference conditions’ those within the geographical area with the lowest AMBI value and highest species richness and diversity. The ‘bad reference conditions’ are 6 for AMBI and 0 for diversity and richness. However, some geographical areas may already be degraded when monitoring has been done in areas assumed to be impacted by human activities. Hence, a second M-AMBI estimate (referred to as M-AMBI-plus) was made using as reference conditions the lowest AMBI value -15%, and the highest richness and diversity +15%, as proposed by Borja et al. (2008) and Borja and Tunberg (2011).

In the main manuscript, AMBI and M-AMBI estimates based on abundance were used. Comparisons of M-AMBI-plus and M-AMBI show that these have differences in status (indicator values), but not in their trend/response to disturbance (Appendix S2: Figure S1, Appendix S2: Figure S2). Comparisons between AMBI based on biomass versus abundance show that biomass-based AMBI has larger variability in response to trawling (Appendix S2: Figure S1). In the individual gradients, biomass-based AMBI also shows more significant changes than abundance-based AMBI (Appendix S2: Figure S2), suggesting it is more sensitive to community changes.

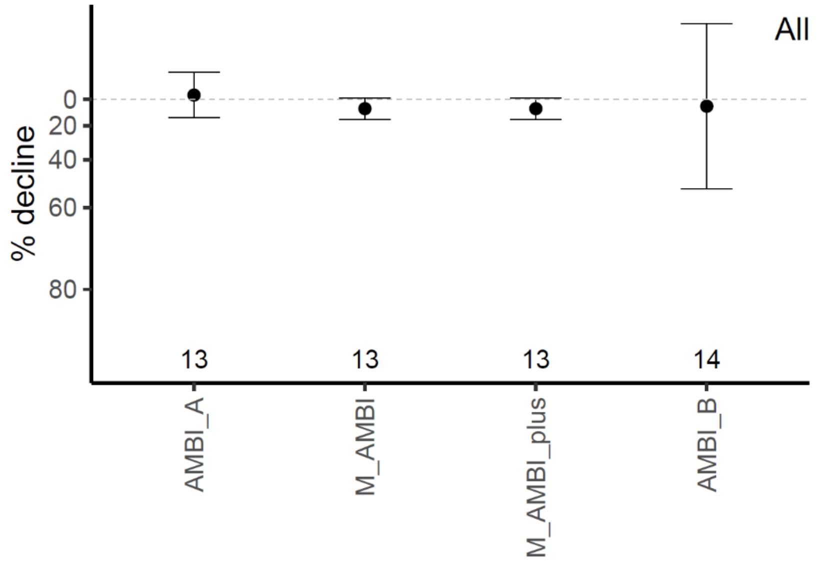

Figure S1. *Sensitivity of the AMBI and M-AMBI indicator response to trawling. Abundance-based AMBI was used to estimate M-AMBI and M-AMBI-plus. Response to bottom trawl fishing disturbance is estimated for all gradients. Dots show the average response across all gradients. A significant effect of trawling is present when the 95% confidence intervals do not overlap with zero. AMBI values are reversed (as low values indicate good state and high values a poor state). A = abundance based, B = biomass based. For other abbreviations, see Table 2 in the main manuscript.*

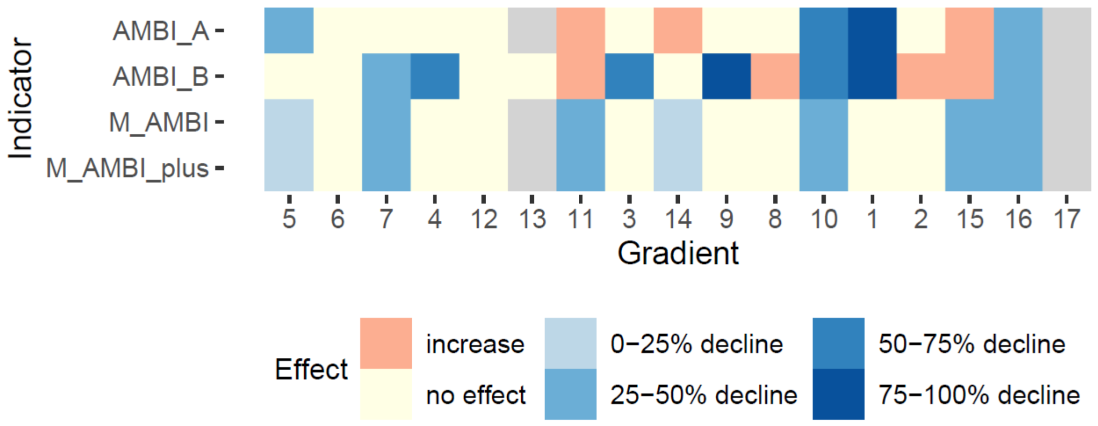

Figure S2. *Comparison of AMBI and M-AMBI responses to all gradients. M-AMBI-plus and M-AMBI provide differences in the status value, but not in their trend/response to disturbance. AMBI trends are reversed (as low values indicate good state and high values a poor state). For numbers and abbreviations, see Table 1 and 2 in the main manuscript.*

Section S2: DKI - Danish Quality Index

DKI (Josefson et al. 2009) is a multi-metric quality index composed of a diversity component represented by the Shannon-diversity (H´) and a sensitivity component, the AMBI (Borja et al. 2000). The two indices are weighted equally in the calculation of DKI which ranges between 0 (poorest ecological/environmental quality) and 1 (highest ecological quality). Calculation of DKI is furthermore adjusted for very low abundances in the sample (equation below). DKI has been inter-calibrated against other Scandinavian quality indices where all indices were tested against each other in various pressure gradients (Josefson et al. 2009). The measurements of each of the two indices (AMBI and Shannon) are scaled relatively to the highest expected value expected at the sampling location calculated as $H´_{observed}$ /$H´_{max}$ and AMBI - $AMBI_{min}$ (the AMBI scales from 0 to 7, where 0 represents the highest ecological quality and therefore the observed value is scale to minimum value).

$$DKI\ v.2 = \frac{\left(1 - \left(\frac{AMBI - AMBImin}{7}\right)\right) + \left(H' \frac{1}{H'max}\right)}{2} \times (1 - \left(\frac{1}{N}\right))$$

As DKI was initially developed for Danish waters, where there are very strong salinity gradients which influences both the species diversity (H´) and the AMBI value, the later version of DKI (DKI v.2) used in this assessment has been normalized to the ambient salinity. Species diversity in the Baltic Sea, and in general, declines almost linearly with declining salinity. In addition, calculation of AMBI has been shown to have dependencies on salinity or other estuarine environmental gradients that parallels the salinity gradient in estuaries. For AMBI, it has been shown that species tolerant to organic enrichment also are relatively more abundant in brackish environments. $AMBI_{min}$ and $H´_{max}$ are therefore normalized to the salinity using an empirical established relationship to salinity obtained from a large number of data sets in across the Baltic Sea (Carstensen et al. 2014). For the data set from the southern Baltic (Gradient 12) and from Gulf of Finland (Gradient 15), salinity was set to 13 and 3 respectively. For the rest of the gradient data sets, salinity was set to 30.

Software tools from AZTI (version 2014) were used to calculated AMBI values (without replication in the input data, i.e., single sample). The values of DKI were estimated for each individual sample and DKI values were afterwards averaged per sampling location.

Section S3: Relative Margalef diversity (D_M')

Margalef diversity was one of the indices tested and applied as part of a multi-index package to be used for benthic habitat quality assessment for the OSPAR Intermediate Assessment (OSPAR 2017, 2018), where it was found to be among the best performing indices in the multi-pressure environment of the Southern North Sea (Van Loon et al. 2018). A way to achieve comparability of Margalef scores that deviate with habitat type was normalization. The methodology is further developed for the OSPAR Quality Status Review (Wijnhoven et al. 2022, OSPAR 2023), applicable with different sampling methodologies and applied in different subregions of the Greater North Sea, now called ‘relative Margalef diversity’.

Margalef's index of diversity (D_M) is given by:

$$D_M = \frac{S-1}{ln\,(N)}$$

where S is the Species Richness and N is the total abundance for each sample. Margalef's index of diversity (D_M) is an absolute measure of diversity. To improve comparability and consider methodological and 'natural' variability, the relative Margalef index of diversity was proposed:

$$D_M{}' = \frac{D_{ass} - D_{bad}}{D_{ref} - D_{bad}}$$

where D_{ass} is the assessed value for the Margalef diversity index, D_{bad} is the Margalef value for a bad ecological state and D_{ref} is the reference value for a good ecological state. D_{ref} is not a pristine reference, but rather a good quality status within reach, considering the current benthic community compositions and species pools, particularly of use for standardization of the assessment methodology, where sampling and laboratory approaches (e.g., identification of species and recording of specimens) might differ between data sets. The value for D_{ref} is estimated based on low pressure observations. Initially D_M is calculated at the level of samples. According to D_M' methodology, usually results for D_M at the sample level are combined with pressure mapping after which the D_{ref} is achieved as a value dependent on the (low) pressure level and data availability (number of samples and years covered) from a case-specific selection of low-pressure data. A percentile value of respectively 75%, 95% or 99% is selected as Dref to be used, from a low pressure data set with SAR values in the 0-0.1 range in case of ≥50, ≥30 and ≥20 samples. In case low pressure data in the range of 0.1-0.5 are used the percentile value of 95% or 99% is selected as Dref in case of ≥50 and ≥30 samples available respectively; optionally the 99% percentile for the SAR 0.5-1 low pressure range only if ≥50 samples available. In case of several potential Dref values available (from the different low pressure categories) the highest value is obtained. Herewith taking 'natural' (and potentially other sources) of variability into account, as there were only a limited number of data available per gradient, and reference values at the level of Broad Habitat Types were only available for the North Sea region, it was decided to use the median of the D_M values with the least pressure for each of the different gradient data sets, as D_{ref}. In all gradient test sets related to fishing pressure, samples with SAR < 0.25 were selected to estimate D_{ref}. Exceptions are gradients 8, 10 and 13, where D_{ref} was based on samples with SAR = 0.0, for gradient 6, SAR = 0.06, and for gradient 14, SAR = 0.5 and gradient 7, where samples < 5% on the relative disturbance frequency gradient were selected. For gradient 15 samples with oxygen levels > 8 mg/l, for gradient 16 samples with total N < 0.1%, and gradient 17 samples with CPI index < 0 were selected to estimate D_{ref}.'

Section S4: BENTIX

BENTIX index (Simboura & Zenetos, 2002) was developed for the purposes of the European Water Framework Directive 2000/60/EC (WFD) and for the assessment of the Ecological Quality Status of benthic macroinvertebrates' communities. It is based on the concept of species sensitivity and uses the relative contribution of two general ecological groups, 'tolerant' and 'sensitive' taxa. The classification of taxa into ecological groups is based on literature review. BENTIX has been tested against a variety of soft-bottom benthic habitats and anthropogenic pressures, such as

eutrophication, organic and chemical pollution. The index has been successfully intercalibrated with other metrics within the Mediterranean geographical intercalibration group (MedGIG) (GIG, 2013), approved by ECOSTAT and established as a national method in Greece and Cyprus for the classification of benthic communities under the WFD.

BENTIX was calculated using the numerical abundance data sets only. In calculating BENTIX, all cephalopods were removed, as well as a number of crustaceans and molluscs usually classified as megafauna, since this index was developed for the benthic macroinvertebrates only. In addition, taxa identified at a high level of taxonomic resolution (e.g., 'bivalves') were not assigned to a specific ecological group, but were accounted to the confidence level of the analysis.

The stations outside the confidence limits of BENTIX (samples with 3 species or less, and/or 6 individuals or less, and/or non-assigned species exceeding 20% of total community abundance) were not included in the analysis. However, an exemption to the latter criterion was applied for Finland's data (gradient 15), since this data set was the only one providing an eutrophication pressure gradient other than Saronikos.

The scale of the BENTIX index ranges from 0 (azoic conditions) to 6 (reference conditions). The same boundaries of the BENTIX ecological status classification could be applied to assess several habitat types of coastal waters, since the index is based on the relative proportions of 'sensitive' and 'tolerant' groups of species. However, the boundaries should be further evaluated across habitat and pressure types, especially in cases where the benthic fauna is naturally dominated by tolerant species. Currently, a modification of High/Good and Good/Moderate boundaries is suggested for the purely muddy habitats (silt and clay particles > 90%), but since the data of granulometry were not available for the data sets provided, such adjustment hasn't been applied.

Section S5: SoS – Sentinels of the Seabed

The indicator Sentinels of the Seabed (SoS) is an ecological indicator developed in the framework of the OSPAR expert groups (BH1 in OSPAR nomenclature) which assesses the status of benthic habitats based on the proportion of the biomass a set of sentinel species as a fraction of the full community (see Serrano *et al.* (2022) for complete details). The indicator determines sentinel species based on two criteria; 1) species that can be frequently found in the natural habitat and 2) species that are sensitive to the studied pressure. To define "frequent or typical species", two different metrics were applied, i) relative contribution of species to intra-habitat similarity between stations sampled in the target habitat within reference condition areas (no disturbance or very low disturbance) using the Similarity Percentages procedure (SIMPER; Clarke 1993), and ii) relative frequency of occurrence for each species within the target habitat under reference conditions. This initial set of "frequent or typical species" is filtered by prioritizing species according to a SoS sensitivity index (species responses to the analysed pressure), avoiding, when possible, tolerant species (i.e., those whose abundance does not show a clear response to the pressure) and always avoiding opportunistic species (i.e., those whose abundance increases with the pressure). SoS sensitivity index is calculated from available classifications of sensitivity to a pressure or pressures group. Previous to WKBENTH2, the SoS indicator was tested (Serrano *et al.*, 2022) using two sensitivity indexes, the BESITO Index (González-Irusta *et al.* 2018) for trawling disturbance, and the

AMBI groups for chemical pollution (Borja *et al.* 2000). Here we also used the AMBI groups to test the SoS indicator for eutrophication and we developed a new Sensitivity Index group based on the longevity classes developed by the WGFBIT (Bolam *et al.* 2014; 2017) to test the case studies based on infauna from the Baltic and North Sea (Appendix S2: Table S1).

Although ideally BESITO should be used for these case studies, the current cover of the BESITO species list (mainly epifaunal species) of the species present in the test data sets evaluated here (mainly infauna) was low (< 40% total richness) or very low (< 20% total richness) so we decided to test a new proxy to sensitivity based on longevity, in the same way as is being tested in WGFBIT (ICES, 2023). For doing this, we assigned a longevity class (<1, 1–3, 3–10,>10 yr) to each taxon (where a taxon could not be assigned to one class, we assigned multiple classes based on fractional scores that summed to 1). Afterwards, we subjectively defined as sensitive species (Sensitivity Index group of 3) all species with a proportion of biomass assigned to the higher longevity class (> 10 years) equal or higher than 0.7. We defined as tolerant (Sensitivity Index group of 2) all species with a proportion of biomass assigned to the higher longevity class lower than 0.7 but higher than this value when the two highest longevity classes were combined (> 3 years). Finally, we defined as opportunistic species (Sensitivity Index group of 1) all species not included in any of the previous categories. This classification is a temporary solution to the lack of BESITO data for these species and cannot be considered final. Therefore, caution is needed when interpreting the results for the SoS indicator based on longevity data. Future work is needed for extending the BESITO Index to a larger list of species or working on a more robust (and further tested) method to assign sensitivity scores based on longevity classes. This proxy for the Sensitivity Index allowed testing of the SoS indicator in a wider group of case studies and is useful to compare the indicator with other approaches. SoS values could not be estimated in gradients with relatively high trawling intensities at the least fished sampling stations.

Once the sentinel species were determined for each gradient (Appendix S2: Table S1) the value of the indicator was computed for each sample as the proportion of these species of the total community biomass.

Table S1. *Sentinel species used to estimate SoS for the different gradient data sets.*

Data Set Code	Sensitivity Index (S.I.)	S.I. Group	Sentinel Species
1	BESITO groups	2	*Anadara demiri*
		3	*Ascidia, Ascidia virgínea, Holothuria tubulosa, Ophiothrix fragilis*
2	BESITO groups	2	*Anadara demiri, Anadara inaequivalvis, Anadara kagoshimensis,Corbula gibba*
		3	*Thyone fusus*
13	BESITO groups	3	*Bathybiaster vexillifer*
		4	*Anthoptilum grandiflorum, Duva florida, Funiculina quadrangularis, Phelliactis* spp., *Heteropolypus sol*
		5	*Actinoscyphia saginata, Stryphnus fortis, Thenea* spp., *Mycale lingua*

9	BESITO groups	3	*Gracilechinus acutus, Lytocarpia myriophyllum , Ophiothrix fragilis, Parastichopus regalis, Actinauge richardi*
10	BESITO groups	3	*Parastichopus tremulus, Araeosoma fenestratum, Hymenodiscus coronata, Nymphaster arenatus*
		4	*Funiculina quadrangularis, Kophobelemnon stelliferum*
		5	*Acanella arbuscula, Asconema setubalense, Pheronema carpenteri, Geodia* sp.
4	Longevity groups	2	*Astropecten* spp, *Hydrozoa., Magelona mirabilis, Nephtys* spp.,<br>*Phaxas pellucidus*
5	Longevity groups	2	*Nephtys, Thyasira, Lumbrineris*
		3	*Pennatula phosphorea, Arctica islandica, Virgularia mirabilis*
11	Longevity groups	2	*Macoma balthica*
12	Longevity groups	2	*Macoma balthica, Scoloplos armiger*
6	Longevity groups	2	*Owenia fusiformis, Parvicardium scabrum*
7	Longevity groups	2	*Ennucula tenuis, Glyceridae, Kurtiella bidentata, Nereididae, Phaxas pellucidus, Polynoidae, Sigalionidae, Synaptidae*
		3	*Thracia convexa*
8	Longevity groups	2	*Glyceridae, Nucula nitidosa, Tellimya ferruginosa*
17	AMBI groups	3	*Ampelisca* sp., *Atylus* sp.<br>*Calyptraea chinensis, Chamelea striatula, Euclymene oerstedi, Eudorella truncatula, Lumbrineris scopa, Maldane glebifex, Metaphoxus fultoni, Musculus costulatus, Nucula* sp.
15	AMBI groups	2	*Marenzelleria* sp.
		3	*Bylgides sarsi*
16	AMBI groups	2	*Aponuphis brementi, Drilonereis filum*<br>*Eunice vittata, Glycera unicornis, Goniada maculata, Harmothoe antilopes, Paralacydonia paradoxa*
		3	*Pilumnus* sp., *Pista cristata, Timoclea ovata*

Section S6: mTDI, pTDI, TDI and mT

Trait-based indices are designed to detect impacts on communities by using semi-quantitative scoring that indicates the potential sensitivity of each species to a given pressure. Here, following on previous studies, a set of five biological traits were selected to characterize potential responses of organisms to physical abrasion (de Juan and Demestre 2012; Bolam and Eggleton, 2014; Foveau et al., 2017; Jac et al. 2020). These traits are (i) position of organisms in the sediment; (ii) feeding mode; (iii) mobility capacity; (iv) adult size and (v) fragility of the structure of organisms. Each trait was subdivided into multiple “modalities” to encompass the range of possible attributes of all taxa.

To allow quantitative analysis, a score was assigned to each modality, varying from low sensitivity (0) to high sensitivity (3)

Trawl disturbance indices (TDI) may be computed on either abundance or biomass, each of which may be log-transformed or not. All outputs are provided following Jac et al. (2020) calculations. mTDI, pTDI, TDI and mT indices are based on species biological traits relevant to this pressure (position, size, mobility, fragility, feeding) and were developed focusing on mega-epifauna (marine invertebrates exceeding 0.5–1 cm in size living on the seafloor surface). In the case of mT, a sixth trait was considered: species protection status. As a result, little to no trait scores were available for endofauna (Foveau et al., 2020). All cephalopods were also removed as they may in some instances largely dominate the community biomass.

The biological trait information may be linked to a given taxa at species, genus and/or family level. When it was not possible to find trait information for some taxa (even when trying to degrade the identification to genus or family level), and if there was less than 75% of the station summed biological metric (abundance or biomass, log or not) that was informed in terms of species traits, the station was removed. As a result, some data sets were only partially informed, and others were fully missing.

In the main manuscript, we used the mTDI, pTDI, TDI and mT results as computed with biomass information as the biomass data covers most gradients with low and high trawl disturbed stations at each gradient (n=9). We compared all versions of the different indicators and found no large differences between indicators (Appendix S2: Figure S3; gradients were included when the log response-ratio could be calculated from at least two stations in each treatment).

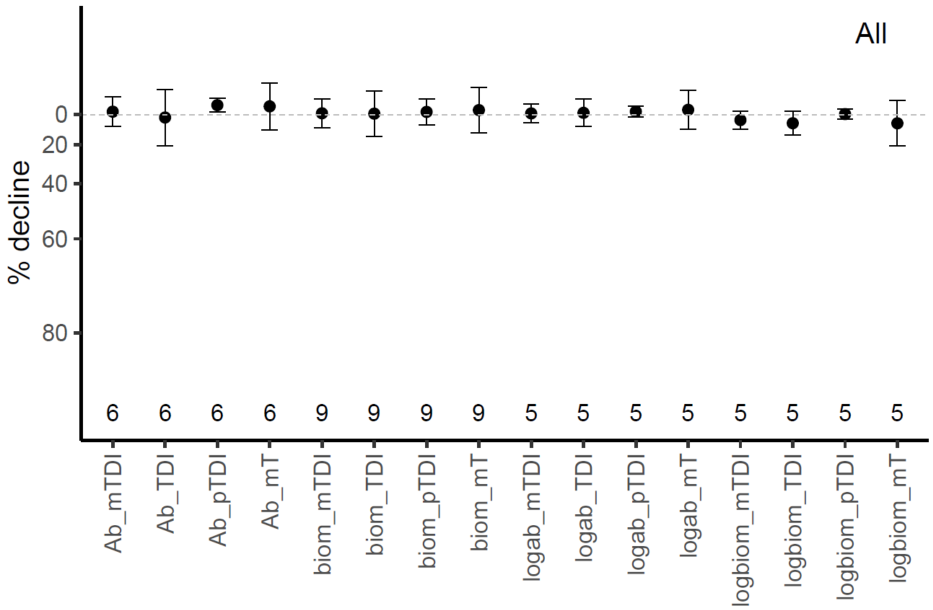

Figure S3. *Response of mTDI, pTDI, TDI and mT indicators to bottom trawl fishing disturbance for all gradients. Dots show the average response across all gradients. A significant effect of trawling is present when the 95% confidence intervals do not overlap with zero. mT values are reversed as low*

values indicate good state and high values a poor state. Ab = abundance based, biom = biomass based, logab = log-transformed abundance, logbiom = log-transformed biomass. For other abbreviations, see Table 2 in the main manuscript.

Section S7: Median longevity and fraction long-lived organisms

The effect of any given rate of trawl mortality on a population will depend on its life-history, whereby populations with low growth, low natural mortality rates and greater longevity have an increased sensitivity to trawling disturbance. Hiddink et al. (2018) showed that the effect of bottom trawling in comparative studies increased with longevity, with a 2-3× larger effect on biota living >10yr than on biota living 1-3yr. This difference was attributed to the slower recovery rates of the longer-lived biota. These results show that the distribution of longevities in a benthic community can be used to estimate the sensitivity to trawling.

Benthic species were linked to a species-by-trait matrix with trait information on longevity (maximum lifespan). Benthic trait information was derived from the ICES working group WGFBIT (ICES, 2023). For most locations, longevity was subdivided into four trait classes (< 1 year, 1–3, 3–10, and > 10 years). For gradient 13, longevity was subdivided into five classes (< 1 year, 1–3, 3–10, 10-50, and > 50 years) based on information from Murillo et al. (2020). For each species-longevity combination, a score of one was assigned to a single class when a species longevity matched a longevity class. Otherwise, fractional scores that summed to one were assigned to multiple longevity classes, following Bolam et al. (2014). From this species-by-longevity matrix, including in some cases higher taxonomic levels, a table of locations by biomass-weighted trait longevity classes was calculated by multiplying the total biomass per species by the longevity score. These were then summed by longevity class and divided by the total biomass of the location to produce a proportional biomass-weighted longevity table for all locations. All cephalopods and fish were removed.

The fraction of long-lived organisms was estimated as the proportional biomass in the most long-lived trait class with organisms.

Median longevity was estimated by converting the biomass by longevity to a cumulative biomass by calculating the biomass proportion with longevity that is smaller than or equal to 1, 3, and 10 years (and 50 years for the gradient 13) in each location. We estimated the biomass–longevity composition using a statistical model, with the cumulative biomass proportions as the response variable and longevity as the predictor variable. Following Rijnsdorp et al. (2018), we used a binomial model where longevity is ln-transformed. We afterwards calculated the median longevity from each statistical model/location. The median longevity describes the longevity in years where the cumulative biomass proportion is 0.5.

Section S8: Biomass, Abundance, Species Richness, Shannon Index, Inverse Simpson, Simpson Index

Total community biomass, abundance and species richness were estimated from the data. Abundance and richness were further used to calculate the Shannon-Wiener, Inverse Simpson and Simpson indices. The diversity indices were estimated in R using the “vegan” package (Oksanen et al. 2020). All cephalopods and fish were removed.

Section S9: Overview of indicators

Of the above indicators, Lm, Lf, TDI, mTDI and pTDI have been specifically developed to assess bottom trawling and DKI, BENTIX, DM’, AMBI and M-AMBI have primarily been used to assess diffuse human pressures such as eutrophication and pollution. Both SoS and mT have been proposed to be more generic by selecting a different set of species/traits depending on the pressure investigated. The other included indicators (R, B, A, H', SI, IS) reflect a benthic community characteristic and are not specific to any pressure.

References

Bolam, S.G., Eggleton, J. D. (2014). Macrofaunal production and biological traits: spatial relationships along the UK continental shelf. Journal of Sea Research, 88, 47–58.

Bolam, S.G., Garcia, C., Eggleton, J., Kenny, A.J., Buhl-Mortensen, L., Gonzalez-Mirelis, G., ... Rijnsdorp, A.D. (2017). Differences in biological traits composition of benthic assemblages between unimpacted habitats. Marine Environmental Research, 126, 1–13.

Borja, A., I. Muxika, 2005. Guidelines for the use of AMBI (AZTI's Marine Biotic Index) in the assessment of the benthic ecological quality. Marine Pollution Bulletin, 50: 787-789.

Borja, A., Franco, J., Perez, V. (2000). A marine biotic index to establish the ecological quality of soft-bottom benthos within European estuarine and coastal environments. Marine Pollution Bulletin 40, 1100–1114.

Borja, A., Dauer, D., Diaz, R., Llanso ,́ R.J., Muxika, I., Rodriguez, J.G., Schaffner, L., (2008). Assessing estu-arine benthic quality conditions in Chesapeake Bay: a comparison of three indices. Ecological Indica-tors 8, 395–403.

Borja, A., Tunberg, B.G. (2011). Assessing benthic health in stressed subtropical estuaries, eastern Florida, USA using AMBI and M-AMBI. Ecol. Indic. 11, 295–303.

Carstensen, J., Andersen, J.H., Gustafsson, B.G., Conley, D. J. (2014). Deoxygenation of the Baltic Sea during the last century. Proceedings of the National Academy of Sciences, 111(15), 5628–5633.

Clarke, K.R. (1993). Non-parametric multivariate analyses of changes in community structure. Australian journal of ecology, 18(1), 117–143.

de Juan, S., & Demestre, M. (2012). A Trawl Disturbance Indicator to quantify large scale fishing impact on benthic ecosystems. *Ecological Indicators*, *18*, 183–190.

Foveau Aurelie, Jac Cyrielle, Llapasset Margaux, Guillerme Chloé, Desroy Nicolas, Vaz Sandrine (2020). Updated biological traits' scoring and protection status to calculate sensitivity to trawling on mega-epibenthic fauna. SEANOE. https://doi.org/10.17882/59517

González-Irusta, J.M., de la Torriente, A., Punzón, A., Blanco, M., Serrano, A. (2018). Determining and map-ping species sensitivity to trawling impacts: the BEnthos Sensitivity Index to Trawling Operations (BESITO). ICES Journal of Marine Science, 75 (5), 1710–1721.

GIG (Geographical Intercalibration Group), 2013. Technical report coastal waters, Mediterranean GIG Benthic Invertebrate fauna, JRC and the benthic macroinvertebrates subgroup. https://circabc.europa.eu/faces/

Hiddink, J. G., Jennings, S., Sciberras, M., Bolam, S. G., Cambiè, G., McConnaughey, R. A., Mazor, T., Hilborn, R., Collie, J. S., Pitcher, R., Parma, A. M., Suuronen, P., Kaiser, M. J., & Rijnsdorp, A. D. (2018). Assessing bottom-trawling impacts based on the longevity of benthic invertebrates. *Journal of Applied Ecology*, *56*, 1075–1084. https://doi.org/10.1111/1365-2664.13278

ICES (2023). Working Group on Fisheries Benthic Impact and Trade-offs (WGFBIT). ICES Scientific Reports. Report. https://doi.org/10.17895/ices.pub.22123193.v1

OSPAR (2017). Intermediate Assessment 2017 - Common indicator. Condition of benthic communities: subtidal habitats of the Southern North Sea. (https://oap.ospar.org/en/ospar-assessments/intermediate-assessment-2017/introduction/ospar-and-intermediate-assessment-2017/).

OSPAR (2018). Common Biodiversity Indicators. Condition of benthic habitat communities (BH2). Generic guidelines for Coordinated Environmental Monitoring Programme (CEMP). OSPAR Agreement 2018-06.

OSPAR (2023). Quality Status Review 2023 - Condition of Benthic Habitat Communities: Margalef diversity in region II (Greater North Sea). (https://oap.ospar.org/en/ospar-assessments/quality-status-reports/qsr-2023/indicator-assessments/).

Jac, C., Desroy, N., Certain, G., Foveau, A., Labrune, C., Vaz, S. (2020a). Part 1: Generic sensitivity indices to measure the effect of trawling on benthic mega-epifauna. Ecological Indicators, 117, 106631.

Josefson, A.B., Blomqvist, M., Hansen, J.L., Rosenberg, R., Rygg, B. (2009). Assessment of marine benthic quality change in gradients of disturbance: comparison of different Scandinavian multi-metric indices. Marine Pollution Bulletin, 58(9), 1263–1277.

Van Loon, W.M.G.M., Walvoort, D.J.J, Van Hoey, G., Vina-Herbon, C., Blandon, A., Pesch, R., Schmidt, P., Scholle, J., Heyer, K., Lavaleye, M., Philips, G., Duineveld, G.C.A., Blomqvist, M. (2018). A regional benthic fauna assessment method for the Southern North Sea using Margalef diversity and reference value modelling. Ecological Indicators 89, 667-679.

Murillo, F.J., Weigel, B., Bouchard Marmen, M., Kenchington, E. (2020). Marine epibenthic functional diversity on Flemish Cap (north-west Atlantic)—Identifying trait responses to the environment and map-ping ecosystem functions. Diversity and Distributions, 26(4), 460–478.

Oksanen, J., Blanchet, F.G., Friendly, M., Kindt R., Legendre, P., ... Wagner, H. (2020). Vegan: Community Ecology Package. R package version 2.5–7. https://CRAN.R-project.org/package=vegan

QSR (2023) (OSPAR). https://www.ospar.org/work-areas/cross-cutting-issues/qsr2023

Rijnsdorp, A.D., Bolam, S.G., Garcia, C., Hiddink, J.G., Hintzen, N.T., van Denderen, P.D., van Kooten, T. (2018). Estimating sensitivity of seabed habitats to disturbance by bottom trawling based on the lon-gevity of benthic fauna. Ecological Applications, 28(5), 1302–1312.

Serrano, A., de la Torriente, A., Punzón, A., Blanco, M., Bellas, J., Durán-Muñoz, P., ... González-Irusta, J. M. (2022). Sentinels of Seabed (SoS) indicator: Assessing benthic habitats condition using typical and sensitive species. Ecological Indicators, 140, 108979.

Wijnhoven, S., Walvoort, D.W.W., Schilder, J. (2022). Condition of benthic habitat communities (BH2-B): Margalef diversity in region II (Greater North Sea). Revision of Annex 3 (CEMP BH2-B by Van Loon, W.M.G.M) of OSPAR agreement 2018-06.
