# Supplementary material for: Complementarity and sensitivity of benthic state indicators to bottom‐trawl fishing disturbance
Source: Ecol Appl. 2024 Oct 12;34(8):e3050. doi: 10.1002/eap.3050 (PMC11610696; doi:10.1002/eap.3050)
Supplement: Supplementary file 3 — Appendix S3: [file EAP-34-e3050-s001.pdf]

Ecological Applications

Appendix S3 – Correlation tables

Complementarity and sensitivity of benthic state indicators to bottom-trawl fishing disturbance

P. Daniël van Denderen, Maider Plaza-Morlote, Sandrine Vaz, Sander Wijnhoven, Angel Borja, Ulla Fernandez-Arcaya, José M. González-Irusta, Jørgen L. S. Hansen, Nikolaos Katsiaras, Andrea Pierucci, Alberto Serrano, Sofia Reizopoulou, Nadia Papadopoulou, Mattias Sköld, Christopher J Smith, Henrik Nygård, Gert Van Hoey, Grete E. Dinesen, Elina A. Virtanen, Aurélien Boyé, Ana García-Alegre, Juan Bellas, Stefan Bolam, Pablo Durán Muñoz, Mar Sacau, Giada Riva, Ellen Kenchington, Saša Raicevich, David Reid, Marie Julie Roux, Jan Geert Hiddink, Sebastian Valanko

Table S1. *Mean correlation of indicators. The mean correlations are computed by averaging all Pearson correlations across all trawl gradient datasets.*

	M-AMBI	DKI	R	Dm'	SI	H'	IS	BEN-TIX	AMBI	B	A	TDI	mTDI	mT	pTDI	Lm	Lf
**DKI**	0.9																
**R**	0.8	0.8															
**Dm'**	0.9	0.7	0.9														
**SI**	0.7	0.7	0.4	0.6													
**H'**	0.9	0.8	0.6	0.7	0.9												
**IS**	0.7	0.7	0.5	0.6	0.9	0.9											
**BENTIX**	0.2	0.2	0.0	0.1	0.1	0.1	0.0										
**AMBI**	0.4	0.5	0.2	0.2	0.2	0.2	0.1	0.6									
**B**	0.4	0.6	0.6	0.4	0.1	0.3	0.2	0.0	0.1								
**A**	0.2	0.5	0.5	0.2	-0.1	0.0	0.0	-0.1	0.1	0.7							
**TDI**	0.1	-0.1	0.1	0.1	0.0	0.1	0.1	-0.2	0.1	0.0	0.0						
**mTDI**	0.1	-0.1	0.0	0.0	0.0	0.1	0.1	-0.2	0.1	0.0	-0.1	1.0					
**mT**	0.0	-0.2	0.0	0.0	0.1	0.1	0.1	-0.1	0.1	0.0	-0.1	0.7	0.8				
**pTDI**	-0.1	-0.4	-0.1	-0.1	-0.1	-0.1	-0.1	0.1	0.0	-0.1	-0.1	0.3	0.3	0.4			
**Lm**	0.1	0.2	0.1	0.1	0.1	0.1	0.1	0.0	0.0	0.2	0.0	0.2	0.2	0.0	0.2		
**Lf**	0.2	0.3	0.2	0.2	0.2	0.2	0.2	0.0	0.1	0.3	0.1	0.2	0.2	0.0	0.2	0.8	
**SoS**	0.0	0.0	0.1	0.0	0.1	0.1	0.1	-0.1	-0.1	0.1	0.0	0.0	0.1	0.1	0.3	0.3	0.3

Table S2. *Standard deviation of correlations across trawl gradient datasets. The standard deviations are determined by calculating Pearson correlations between two indicators for each dataset. Subsequently, a standard deviation is computed across datasets based on these correlation values. A high standard deviation indicates greater variability in the correlations between gradient datasets.*

	**M-AMBI**	**DKI**	**R**	**Dm'**	**SI**	**H'**	**IS**	**BEN-TIX**	**AMBI**	**B**	**A**	**TDI**	**mTDI**	**mT**	**pTDI**	**Lm**	**Lf**
**DKI**	0.1																
**R**	0.2	0.2															
**Dm'**	0.1	0.1	0.0														
**SI**	0.2	0.3	0.3	0.2													
**H'**	0.1	0.2	0.3	0.2	0.1												
**IS**	0.2	0.3	0.3	0.2	0.1	0.1											
**BENTIX**	0.5	0.6	0.5	0.5	0.6	0.5	0.5										
**AMBI**	0.4	0.4	0.5	0.5	0.6	0.5	0.5	0.4									
**B**	0.5	0.2	0.3	0.4	0.4	0.4	0.4	0.5	0.6								
**A**	0.6	0.5	0.4	0.5	0.6	0.6	0.5	0.5	0.6	0.3							
**TDI**	0.6	0.7	0.4	0.4	0.5	0.6	0.5	0.5	0.6	0.5	0.6						
**mTDI**	0.6	0.7	0.5	0.5	0.6	0.6	0.5	0.5	0.6	0.6	0.6	0.0					
**mT**	0.5	0.6	0.4	0.4	0.5	0.5	0.4	0.5	0.6	0.5	0.6	0.5	0.4				
**pTDI**	0.6	0.6	0.5	0.5	0.5	0.6	0.5	0.6	0.6	0.5	0.5	0.7	0.7	0.5			
**Lm**	0.5	0.5	0.4	0.4	0.4	0.5	0.4	0.5	0.5	0.4	0.3	0.6	0.6	0.6	0.5		
**Lf**	0.4	0.4	0.4	0.4	0.4	0.4	0.4	0.5	0.5	0.4	0.4	0.6	0.6	0.6	0.5	0.2	
**SoS**	0.6	0.5	0.5	0.6	0.4	0.5	0.4	0.5	0.5	0.5	0.4	0.6	0.6	0.5	0.5	0.5	0.4
