# Supplementary material for: Complementarity and sensitivity of benthic state indicators to bottom‐trawl fishing disturbance
Source: Ecol Appl. 2024 Oct 12;34(8):e3050. doi: 10.1002/eap.3050 (PMC11610696; doi:10.1002/eap.3050)

Ecological Applications

Appendix S4 – meta-analysis station selection

Complementarity and sensitivity of benthic state indicators to bottom-trawl fishing disturbance

P. Daniël van Denderen, Maider Plaza-Morlote, Sandrine Vaz, Sander Wijnhoven, Angel Borja, Ulla Fernandez-Arcaya, José M. González-Irusta, Jørgen L. S. Hansen, Nikolaos Katsiaras, Andrea Pierucci, Alberto Serrano, Sofia Reizopoulou, Nadia Papadopoulou, Mattias Sköld, Christopher J Smith, Henrik Nygård, Gert Van Hoey, Grete E. Dinesen, Elina A. Virtanen, Aurélien Boyé, Ana García-Alegre, Juan Bellas, Stefan Bolam, Pablo Durán Muñoz, Mar Sacau, Giada Riva, Ellen Kenchington, Saša Raicevich, David Reid, Marie Julie Roux, Jan Geert Hiddink, Sebastian Valanko

Table S1. Minimum, mean, and maximum trawling intensities of the least and most heavily fished stations used in the meta-analysis.

#	Location	Least fished (min, median, max)	Most fished (min, median, max)	Trawling intensity unit
1	Adriatic Sea – Italian EEZ	0, 0, 0 (n=3)	2.71, 4.72, 10.60 (n=5)	SAR per year
2	Adriatic Sea – Italian EEZ	0, 0, 0 (n=4)	7.93, 13.90, 18.80 (n=5)	SAR per year
3	North Sea - Dutch EEZ	0.06, 0.16, 0.25 (n=2)	2.64, 3.08, 3.61 (n=5)	SAR per year
4	North Sea - Dogger Bank	0.14, 0.22, 0.27 (n=4)	3.68, 4.31, 4.94 (n=2)	SAR per year
5	North Sea - Fladen Ground	0.14, 0.24, 0.35 (n=4)	1.18, 1.64, 3.00 (n=5)	SAR per year
6	North Sea - Long Forties	0.06, 0.21, 0.35 (n=3)	1.41, 1.48, 1.55 (n=2)	SAR per year
7	North Sea - Silver Pit	1.75, 3, 4.25 (n=2)	29, 56, 99 (n=3)	Relative frequencies of trawling disturbance on a linear scale
8	North Sea – Thames	0, 0, 0 (n=3)	54, 77, 100 (n=3)	Relative frequencies of trawling disturbance on a linear scale
9	Northern Iberian Coast	0, 0.02, 0.07 (n=3)	3.57, 5.69, 10.4 (n=5)	SAR per year
10	Northern Iberian Coast	0, 0.08, 0.23 (n=5)	14.7, 17.6, 21.1 (n=5)	SAR per year
11	Baltic Sea - Gotland	0, 0, 0 (n=4)	4.67, 5.01, 5.35 (n=2)	SAR per year
12	Baltic Sea – Polish EEZ	0.04, 0.10, 0.15 (n=2)	2.02, 3.90, 7.14 (n=5)	SAR per year
13	NW Atlantic - Flemish Cap	0, 0, 0 (n= 6)	1.54, 4.07, 12.2 (n=5)	Number of VMS pings by square km
14	Irish Sea - Sellafield	0.5, 1.6, 2.7 (n=2)	8.8, 10.2, 11.9 (n=5)	SAR per year

Figure S1. We conducted a sensitivity test on the meta-analysis results by systematically reducing the number of sampling stations included in both the least and most fished groups. Panel (a) contains a maximum of 4 sampling stations, panel (b) contains 3, and panel (c) contains 2 in each treatment group. The reduction process involved removing stations with the lowest intensity from the high-fished group, while conversely, eliminating stations with the highest intensity from the low-fished group. We made an exception for all sampling stations in the low treatment group with zero fishing intensity, as these were retained in each analysis.

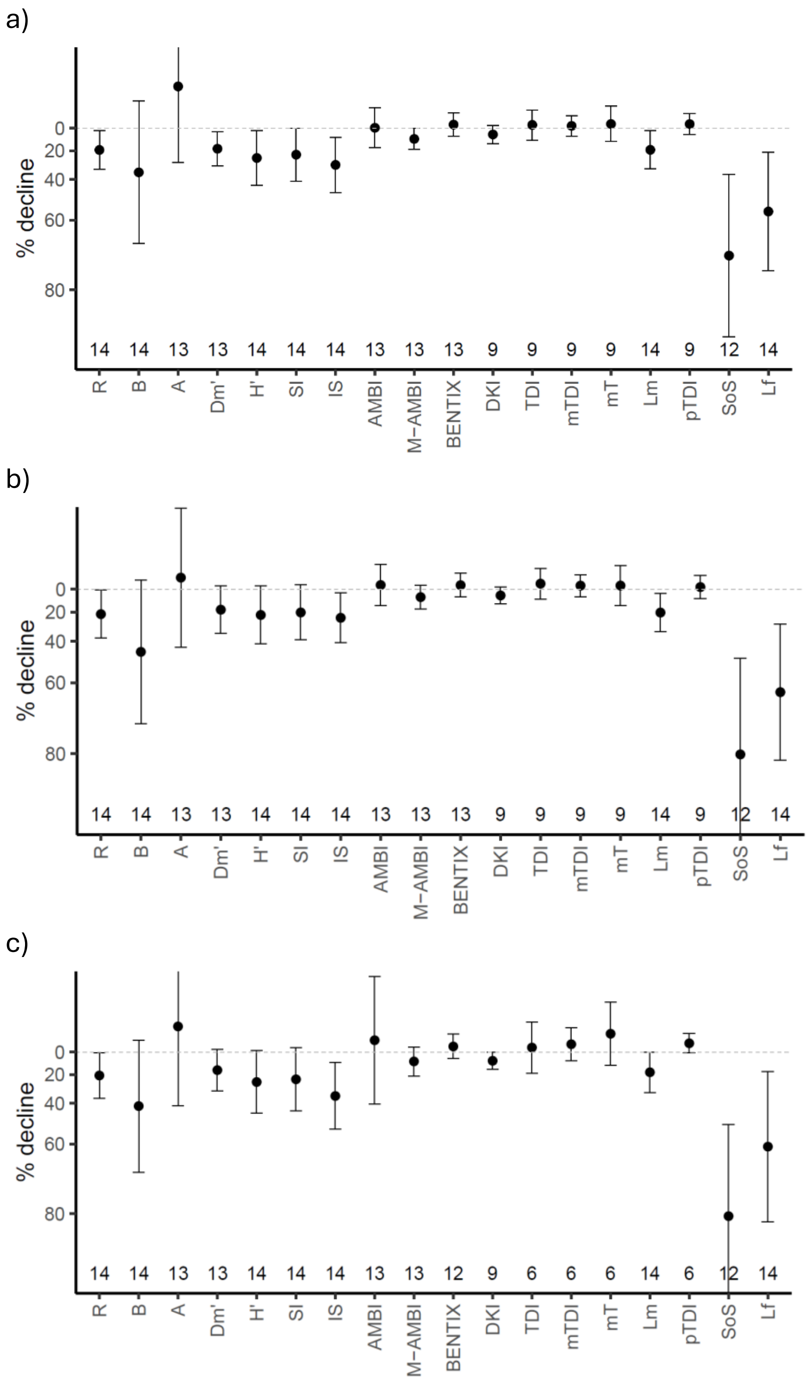

Supplement: Supplementary file 4 — Appendix S4: [file EAP-34-e3050-s002.pdf]
